# Low Frequency Variants, Collapsed Based on Biological Knowledge, Uncover Complexity of Population Stratification in 1000 Genomes Project Data

**DOI:** 10.1371/journal.pgen.1003959

**Published:** 2013-12-26

**Authors:** Carrie B. Moore, John R. Wallace, Daniel J. Wolfe, Alex T. Frase, Sarah A. Pendergrass, Kenneth M. Weiss, Marylyn D. Ritchie

**Affiliations:** 1 Center for Human Genetic Research, Department of Molecular Physiology and Biophysics, Vanderbilt University, Nashville, Tennessee, United States of America; 2 Center for Systems Genomics, Department of Biochemistry and Molecular Biology, The Pennsylvania State University, Eberly College of Science, The Huck Institutes of the Life Sciences, University Park, Pennsylvania, United States of America; 3 Department of Anthropology, The Pennsylvania State University, University Park, Pennsylvania, United States of America; Wellcome Trust Sanger Institute, United Kingdom

## Abstract

Analyses investigating low frequency variants have the potential for explaining additional genetic heritability of many complex human traits. However, the natural frequencies of rare variation between human populations strongly confound genetic analyses. We have applied a novel collapsing method to identify biological features with low frequency variant burden differences in thirteen populations sequenced by the 1000 Genomes Project. Our flexible collapsing tool utilizes expert biological knowledge from multiple publicly available database sources to direct feature selection. Variants were collapsed according to genetically driven features, such as evolutionary conserved regions, regulatory regions genes, and pathways. We have conducted an extensive comparison of low frequency variant burden differences (MAF<0.03) between populations from 1000 Genomes Project Phase I data. We found that on average 26.87% of gene bins, 35.47% of intergenic bins, 42.85% of pathway bins, 14.86% of ORegAnno regulatory bins, and 5.97% of evolutionary conserved regions show statistically significant differences in low frequency variant burden across populations from the 1000 Genomes Project. The proportion of bins with significant differences in low frequency burden depends on the ancestral similarity of the two populations compared and types of features tested. Even closely related populations had notable differences in low frequency burden, but fewer differences than populations from different continents. Furthermore, conserved or functionally relevant regions had fewer significant differences in low frequency burden than regions under less evolutionary constraint. This degree of low frequency variant differentiation across diverse populations and feature elements highlights the critical importance of considering population stratification in the new era of DNA sequencing and low frequency variant genomic analyses.

## Author Summary

Low frequency variants are likely to play an important role in uncovering complex trait heritability; however, they are often continent or population specific. This specificity complicates genetic analyses investigating low frequency variants for two reasons: low frequency variant signals in an association test are often difficult to generalize beyond a single population or continental group, and there is an increase in false positive results in association analyses due to underlying population stratification. In order to reveal the magnitude of low frequency population stratification, we performed pairwise population comparisons using the 1000 Genomes Project Phase I data to investigate differences in low frequency variant burden across multiple biological features. We found that low frequency variant confounding is much more prevalent than one might expect, even within continental groups. The proportion of significant differences in low frequency variant burden was also dependent on the region of interest; for example, annotated regulatory regions showed fewer low frequency burden differences between populations than intergenic regions. Knowledge of population structure and the genomic landscape in a region of interest are important factors in determining the extent of confounding due to population stratification in a low frequency genomic analysis.

## Introduction

In the field of human genetics research, there has been increasing interest in the role of low frequency variation in complex human disease (defined in this text as variants with a minor allele frequency between 0.5%–3%). This is in many ways a response to changing technology, but more importantly a response to the inability to completely explain heritability in common complex diseases and recognition of the true multifactorial mechanisms of genetic inheritance [1]. Since low frequency variants are likely essential in understanding the etiology of common, complex traits, it is critical to elucidate the genetic architecture and population substructure of low frequency variants for future work in this field. Factors such as rapid population growth and weak purifying selection have allowed ancestral populations to accumulate an excess of low frequency variants across the genome. This affects genomic analyses in two ways: proportion of deleterious versus neutral variation expected in low frequency variants and population stratification.

It has been suggested that slightly deleterious single nucleotide variants (SNVs) subjected to weak purifying selection are major players in common disease susceptibility [2], [3]. For example, Nelson et al. found that in 202 drug target genes, 2/3 of the low frequency variants were nonsynonymous mutations. This is a much higher ratio than found for common variants, and reflect the expected proportion given random mutation and degenerate coding. This ratio also suggests low frequency variants are only weakly filtered by selection [2], [4]. In addition, low frequency variants represent a considerable proportion of the genome due to recent explosive population growth [3]. Gorlov estimates up to 60% of SNVs in the genome are variants with an allele frequency <5% [5]. Since the allele frequency distribution is skewed towards more low frequency variants, a higher number of low frequency deleterious variants are expected. Subsequently, low frequency variants appear to be enriched for functional variation, including protein coding changes and altered function [6].

Further, low frequency variants exhibit extreme population stratification. Demonstrating the magnitude of low frequency population stratification between two populations, Tennessen et al. identified more than 500,000 SNVs using 15,585 protein-coding genes from 2,440 individuals. Of these SNVs, 86% had a MAF<0.5% and 82% were population specific between European Americans and African Americans [3]. Low frequency allele sharing between populations on the same continent can be between 70% and 80%. In contrast, low frequency allele sharing between populations on different continents can be lower than 30% and variants are often unique to a single population. This extreme geographic stratification can lead to higher false positives and difficulty in replicating associations across genetic studies when not considered as part of the experimental design for low frequency SNV analyses [6].

To study the “landscape” of low frequency variant stratification across populations, we grouped low frequency variants across pertinent genome-wide biological features in a series of pairwise population comparisons across multiple ancestries. We define the boundaries of grouping by features, which consist of genomic regions (one or many) that belong to a genomic category, for example, a gene or a set of genes in a pathway. Methods that aggregate variants have been shown to be much more powerful than single-variant association testing for low frequency variants [7]–[10], and thus are reliable to detect population stratification. Our collapsing method, BioBin, provides the opportunity to cast a broader net and uncover stratification across meaningful elements such as genes, pathways, and evolutionary conserved regions by aggregating low frequency variants based on expert biological knowledge.

Herein we have applied BioBin to individuals from 1000 Genomes Project Phase I data; we defined “cases” and “controls” randomly between exhaustive pairwise population comparisons. Our goal was to identify features across the genome with differences in low frequency burden between populations; specifically, to look for aggregate differences in low frequency variation between populations, not to detect individual population-specific variants. We show that BioBin is effective in identifying differences in low frequency variant burden centered on biological criteria and highlights the considerable differences in low frequency variants across ancestry groups. These results further emphasize the critical importance of considering low frequency population substructure in future rare and low frequency variant analyses.

## Results

### Low frequency variant burden analysis in 1000 Genomes Project data

We applied BioBin to whole-genome population data using the 1000 Genomes Project Phase I data. The populations, sample sizes, and total number of loci, variants, low frequency variants, and private variants are listed in Table 1. Although the Iberian population (IBS) is listed in Table 1, this population was not used in the analyses presented in this paper. There was not a sufficient sample size to meet our low frequency criteria (N = 14, MAF cutoff = 0.03).

**Table 1:** Phase I 1000 Genomes Project data characteristics.

Continental Group	POP	POPULATION	N	REL	TOTAL LOCI	TOTAL VARIANTS	LOW FREQ VARIANTS	PRIVATE VARIANTS
African descent (AFR)	ASW	HapMap African ancestry individuals from SW US	61	5	18819173	18762530	7948290	1059215
	LWK	Luhya individuals	97	10	19936728	19857956	8781777	2600039
	YRI	Yoruba individuals	88	0	18022152	17926400	7328288	1032847
Asian descent (EAS)	CHB	Han Chinese in Beijing	97	16	10566371	10292757	3673350	860493
	CHS	Han Chinese South	100	16	10547019	10251069	3872508	1102270
	JPT	Japanese individuals	89	17	10368186	10063756	3535488	1233969
European descent (EUR)	CEU	CEPH individuals	87	0	11198921	10994490	4028071	520730
	FIN	HapMap Finnish individuals from Finland	93	0	11005104	10799742	3549441	524199
	GBR	British individuals from England and Scotland	89	3	11411688	11212275	4064515	576664
	IBS	Iberian populations in Spain	14	0	8424366	8155987	0	129800
	TSI	Toscan individuals	98	0	11858607	11668150	4502592	818043
Spanish/Mexican descent (AMR)	CLM	Colombian in Medellin, Colombia	60	1	13869201	13753047	6063724	729009
	MXL	HapMap Mexican individuals from LA California	66	7	12929352	12788406	5322835	840056
	PUR	Puerto Rican in Puerto Rico	55	0	14066653	13958200	6266201	561551

Fourteen populations released in the Phase I 1000 Genomes Project data release, including the continental group, population abbreviation (POP), short description of each population (POPULATION), number of individuals (N), number of cryptically related individuals dropped in final analyses (REL), total number of loci, variants, low frequency variants (MAF< = 0.03), and private variants. Only autosomal variants were considered. The total loci column refers to the number of variant lines in the VCF file, but not all of these lines contain binnable variants, due to filtering and missing data.

**Figure 1:**
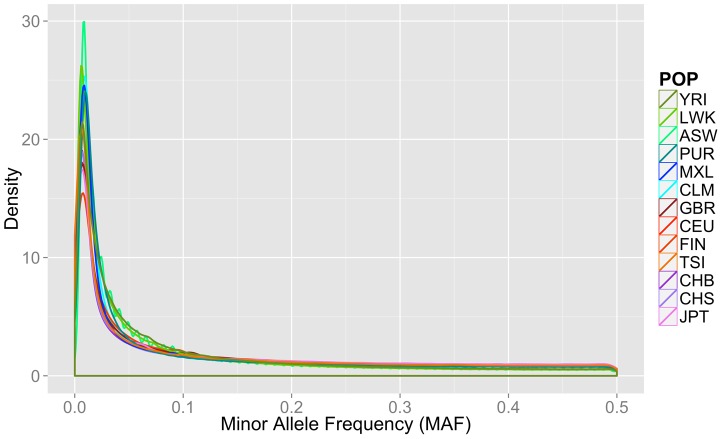
Minor allele frequency distribution on autosomal chromosomes for 13 1000 Genomes Project Phase I populations. Groups are color coordinated by continental ancestry: greens = African descent (YRI, LWK, ASW); blues = Mexican/Spanish descent (PUR, CLM, MXL); orange/reds = European descent (GBR, FIN, CEU, TSI); and pink/purple colors = Asian descent (JPT, CHB, CHS). The populations of African descent have the highest proportion of low frequency variation.

In addition to the differences in overall magnitude of variation seen in Table 1 between these population groups, there were also differences in the distribution of this variation. In Figure 1, we present an allele frequency density distribution plot of all autosomal chromosomes for all 13 populations. African descent populations have the highest density of low frequency variation. Others have found a similar trend genome-wide [11]. In general, the African ancestral populations not only have more variants overall than other ancestral groups (see Table 1), these populations also have a higher distribution of low frequency variants than other ancestral groups (see Figure 1).

Although low coverage next generation sequence data is prone to errors, we found no evidence that sequence technology led to differential bias in a way that could explain the trends found in this paper (Text S1, Table S1, Figures S1, S2).

### Investigation of allele sharing

We investigated sample-relatedness with respect to common and low frequency variants using both identity-by-descent (IBD) and identity-by-state (IBS) estimations, and in each analysis, we found evidence of increased relatedness in ASW (African ancestry, USA), CHB (Han Chinese Beijing, China), CHS (Han Chinese Shanghai, China), CLM (Medellin, Columbia), GBR (England and Scotland), JPT (Japan), LWK (Luhya, Kenya), and MXL (Mexican Ancestry, California). We performed iterative IBD calculations in plink to eliminate related individuals from continental groups. Seventy-five individuals of 1080 total individuals were parsimoniously removed to achieve a pi_hat< = 0.3 in each continental population. The remaining 1,005 individuals were used for the binning analyses presented in this paper.

**Figure 2:**
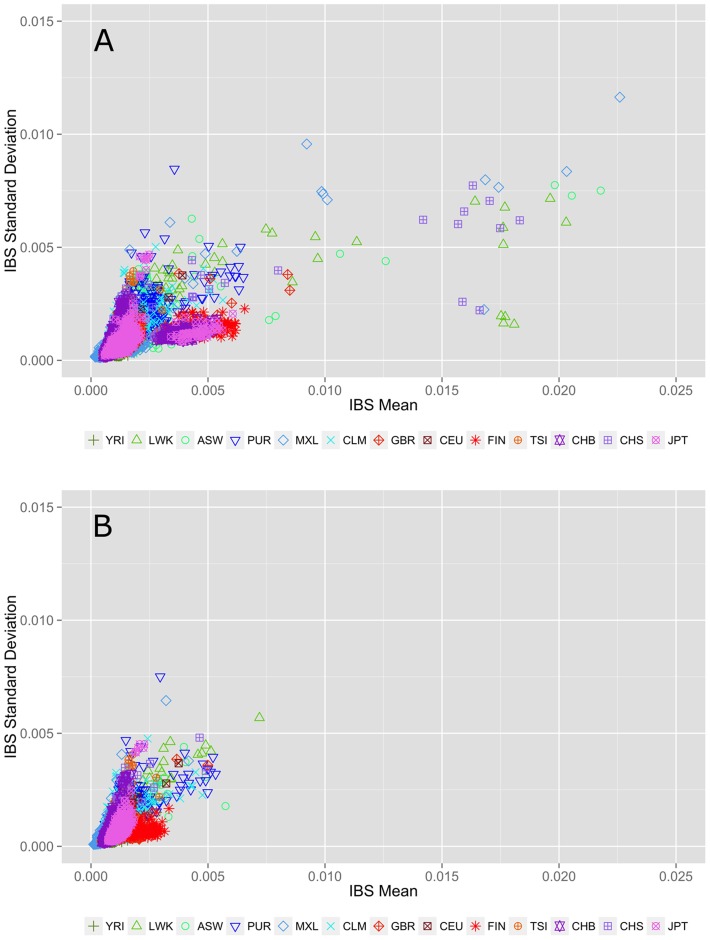
Within population identity-by-state (IBS) estimations A) before and B) after removing individuals with cryptic relatedness. The x-axis represents the IBS mean for low frequency variants averaged over 22 autosomal chromosomes. The y-axis corresponds to the standard deviation of IBS scores across 22 autosomal chromosomes. The colors and point types correspond to each population; color schemes correspond to general ancestry groups as defined for Figure 1. Each point represents a population pairwise IBS calculation (i.e. YRI-YRI, not YRI-CEU). Identifying and excluding related individuals removes the outliers seen in the top plot.

An alternate allele sharing method described by Abecasis et al. uses IBS rather than IBD to review allele sharing [12], [13]. In the case of low frequency or rare variants, IBS approximates IBD. Figure 2 shows within population IBS for all 13 populations for variants with a MAF<3%, where each point represents a pairwise IBS calculation within the same population (i.e. YRI-YRI but not YRI-CEU). In Figure 2A, the pairs with average IBS calculations that fall outside of the cluster are cryptically related individuals with increased allele sharing. Figure 2B shows the IBS calculations after removing 75 individuals with cryptic relatedness. Complete details of these and additional sample-relatedness analyses are available in Text S2, Figure S3, Figure S4, and Figure S5.

### Genomic feature exploration

**Figure 3:**
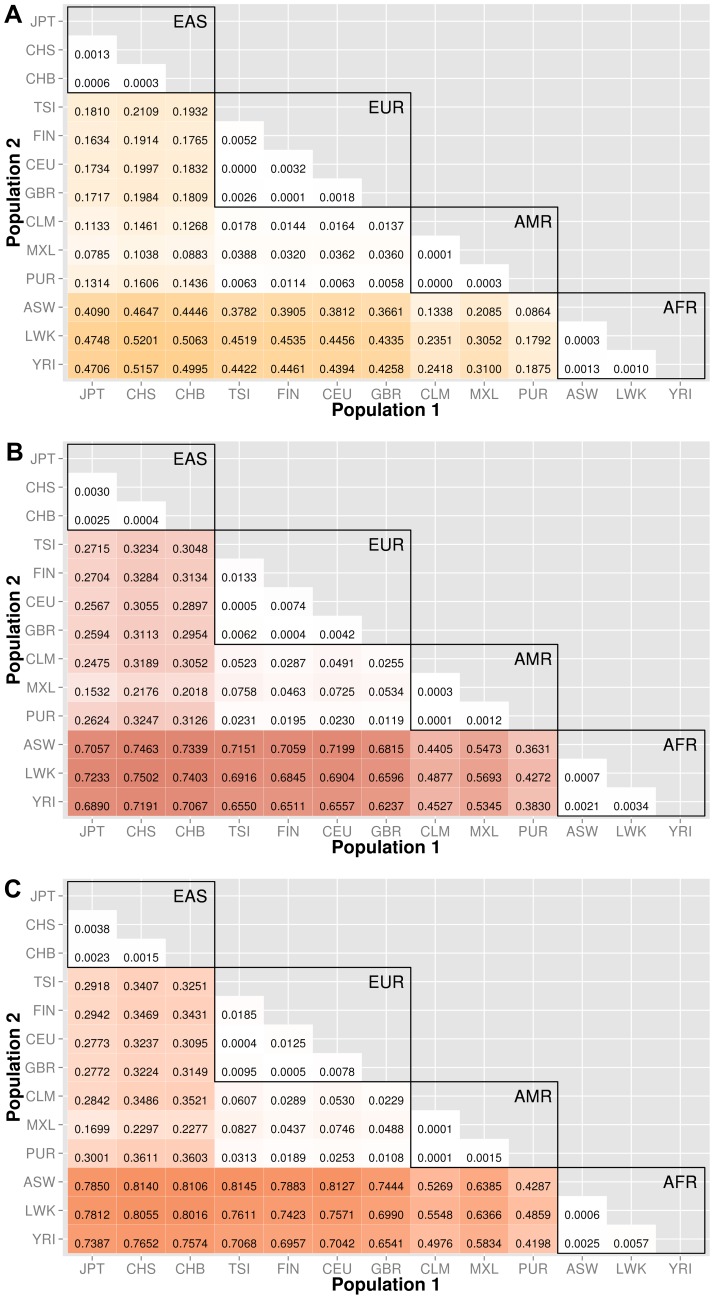
Proportion of significantly different bins in A) gene exon, B) gene intron, and C) intergenic regions. The abbreviations for the each population on found on the x and y-axes. The numbers in each block and the color intensity [0,1] indicate the proportion of significant bins (after Bonferroni correction) for the 1000 Genomes populations on each axis, where the darker the color, the higher the proportion of significant bins. In general, the x-axis is organized with African descent populations on the far right and increasing differentiation with regard to low frequency burden towards the left (i.e. populations of Asian descent have the highest proportion of significant bins compared to African descent groups). The proportion of significant bins across all population comparisons increases from coding (A) to noncoding (B) and finally intergenic (C) regions.

**Figure 4:**
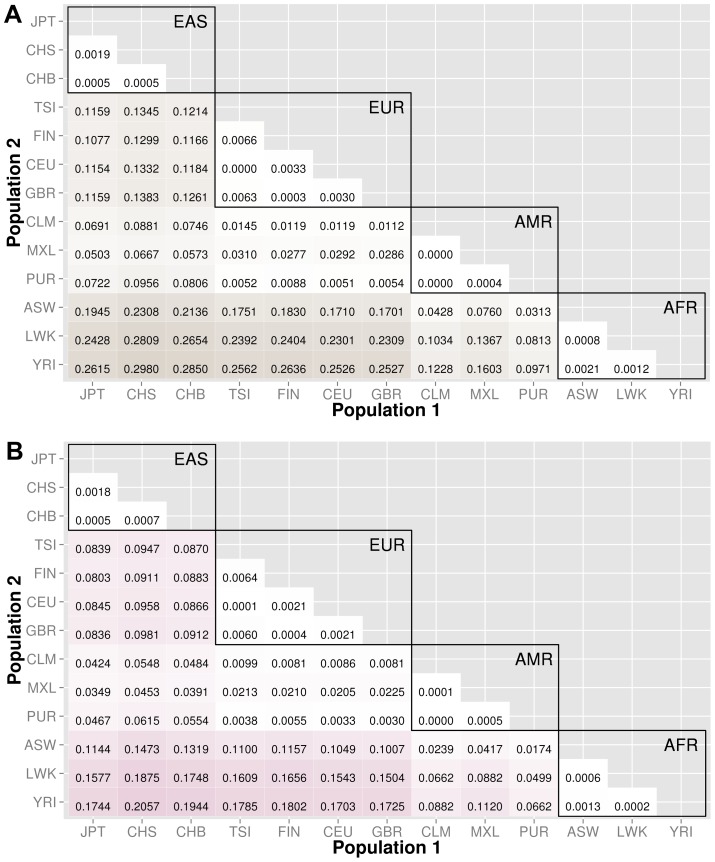
Proportion of significantly different bins for gene-exon filters: A) nonsynonymous and B) predicted deleterious variants. The abbreviations for the each population on are on the x and y-axes. The numbers in each block and the color intensity [0,1] indicate the proportion of significant bins (after Bonferroni correction) for the 1000 Genomes populations on each axis, where the darker the color, the higher the proportion of significant bins. In general, the x-axis is organized with African descent populations on the far right and increasing differentiation with regard to low frequency burden towards the left (i.e. populations of Asian descent have the highest proportion of significant bins compared to African descent groups). Filtering gene exon regions by mutation type and predicted functional significance lead to smaller bins and overall greatly reduced proportions of significance.

**Figure 5:**
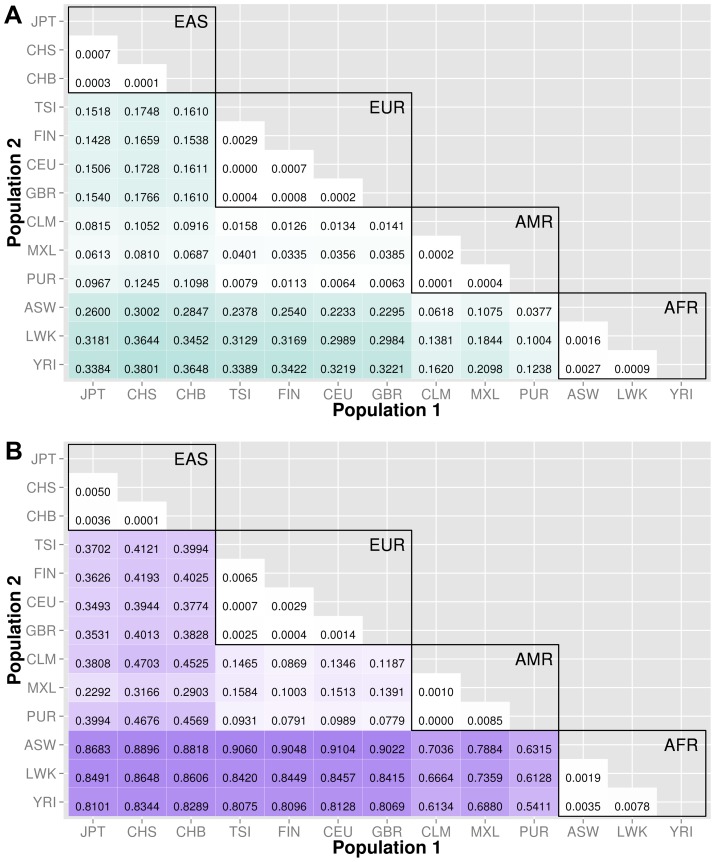
Proportion of significantly different bins in A) ORegAnno regulatory and B) pathway feature analysis. The abbreviations for the each population on are on the x and y-axes. The numbers in each block and the color intensity [0,1] indicate the proportion of significant bins (after Bonferroni correction) for the 1000 Genomes populations on each axis, where the darker the color, the higher the proportion of significant bins. In general, the x-axis is organized with African descent populations on the far right and increasing differentiation with regard to low frequency burden towards the left (i.e. populations of Asian descent have the highest proportion of significant bins compared to African descent groups). From more conserved regulatory regions to relatively large binned pathways, Figure 5A shows conservation in comparison to genic regions (Figure 3) and Figure 5B shows occasionally very high proportions of significant bins in parent pathway bins in comparison to genic regions (Figure 3).

**Figure 6:**
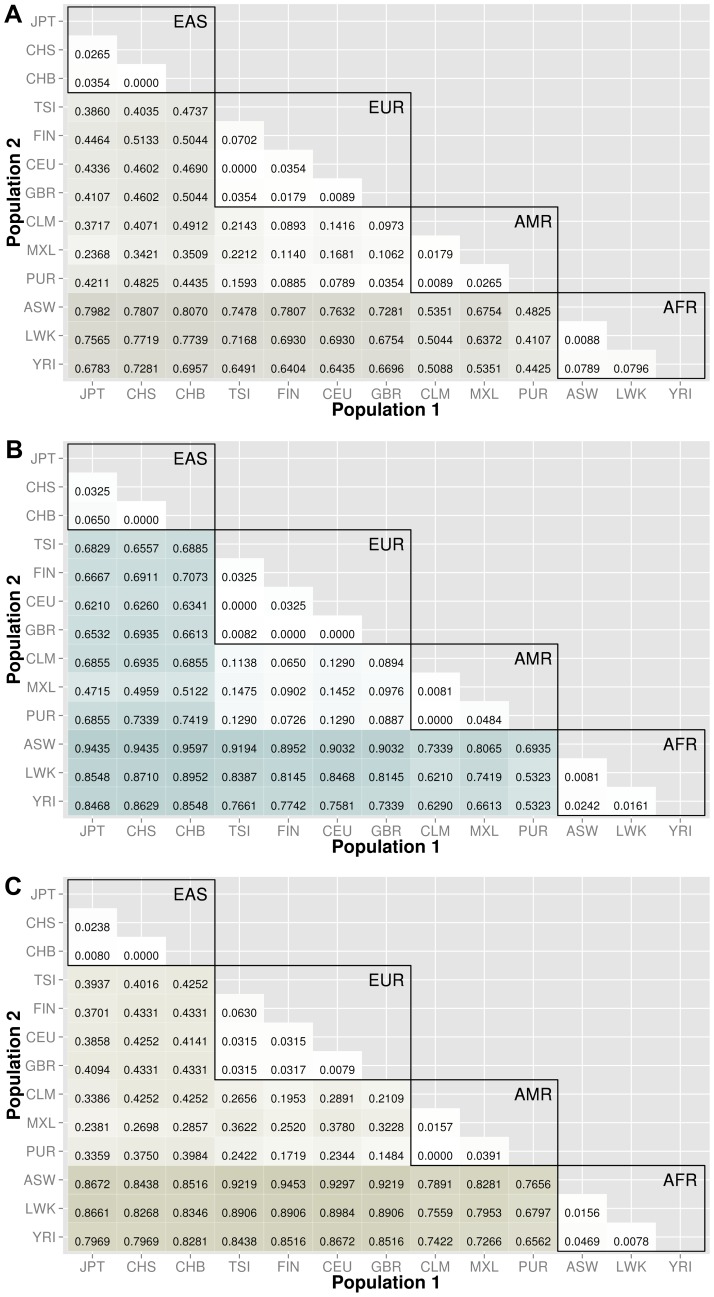
Proportion of significantly different bins in natural selection analysis by region of identification: A) AFR continental group, B) EAS continental group, and C) EUR continental group. The abbreviations for the each population on are on the x and y-axes. The numbers in each block and the color intensity [0,1] indicate the proportion of significant bins (after Bonferroni correction) for the 1000 Genomes populations on each axis, where the darker the color, the higher the proportion of significant bins. In general, the x-axis is organized with African descent populations on the far right and increasing differentiation with regard to low frequency burden towards the left (i.e. populations of Asian descent have the highest proportion of significant bins compared to African descent groups). The regions of natural selection, particularly negative selection, are often accompanied by excess low frequency variants. As world populations evolved, selective forces were often unique and location specific. Therefore, the evolution of low frequency variants compared across world populations can be markers of past selective events. Populations within a continental group are very similar and we see high proportions of statistically significant bins between populations of different continental groups.

Knowledge of population substructure in low frequency variants is critical for genomic studies. We applied BioBin to test for low frequency (MAF≤0.03) variant burden differences between 13 populations from the 1000 Genomes Project across different genomic features: genes (intronic and exonic variants, filtered nonsynonymous and predicted damaging variants), intergenic regions, ORegAnno annotated regulatory regions, pathways, pathway-exons, evolutionary conserved regions, and regions considered to be under natural selection. Results are shown in Figure 3, Figure 4, Figure 5, Figure 6, Figure S6, and Figure S7. In each matrix plot, we have indicated the proportion of significant bins (after Bonferroni correction) out of the total number of bins generated between two populations. The color intensity represents the proportion of total bins that were significant [0,1]. Overall, there are large differences across populations with regard to low frequency variant burden and the distribution of low frequency variants is not random across the genome. The magnitude of stratification corresponds to the mutational landscape of the region.

### Coding and noncoding regions

We chose NCBI Entrez to provide the boundaries for gene regions and created a custom role file of intron and exon boundaries using data provided from UCSC Genome Browser [14]. In Figure 3, the top matrix corresponds to bins created using gene-exon boundaries, the middle matrix corresponds to bins created using gene-intron boundaries, and the bottom matrix corresponds to bins created using regions between genes (intergenic). The values and color intensity within each block represent the proportion of significant bins after Bonferroni correction out of the total number of bins generated between two populations.

The coding regions show a trend of a lower proportion of significant bins with low frequency variant burden differences than either the intron or intergenic bins. For example, in the CEU (Northern/Western European Ancestry, USA)−YRI (Yoruba African) comparison, approximately 44% of the gene-exon bins had significant differences in low frequency variant burden. In contrast, the noncoding region bins, gene-introns and intergenic bins had 66% and 70% of bins with significant differences in low frequency variant burden. The coding regions appear to be under more constraint across populations than noncoding regions. Comparing only the noncoding regions, introns tend to have slightly fewer variation differences than intergenic bins, most likely because introns are by default nearest neighbors to the selective pressures on coding regions.

We filtered the gene-exon bins using annotations from the Variant Effect Predictor Software (VEP) [15]. We created gene bins with only nonsynonymous variants and a second analysis using only predicted damaging variants annotated by SIFT or PolyPhen2 [15]–[17]. The results in Figure 4 indicate that these potentially functional and significant changes are even more conserved between populations than coding regions (Figure 3A).

### ORegAnno annotated regions

We used ORegAnno (Open Regulatory Annotation database) to define regulatory region boundaries for the bin analysis. The top matrix of Figure 5 shows the 78 population comparisons for the ORegAnno regulatory feature analysis.

In comparison to Figure 3, the annotated regulatory regions have fewer significant bins. For example, in gene-exon analysis shown in Figure 3, approximately 44% of the ASW-CHB gene-exon bins contained significant differences in low frequency burden. However, in Figure 5, only 28% of the ASW-CHB annotated regulatory bins contained significant differences in low frequency burden. This trend is consistent across the matrix of population comparisons; regulatory regions have fewer significant bins than the coding or noncoding features of the same population comparison.

### Pathway and group features

Several biological pathway and group sources from LOKI (the Library of Knowledge Integration, which is described in detail in the methods) were used to generate low frequency variant bins; including, PFAM, KEGG, NetPath, PharmGKB, MINT, GO, dbSNP, Entrez, and Reactome. The Figure 5B shows the 78 population comparisons for the pathway group feature analysis.

Of all of the feature analyses, pathway bins consistently show the highest proportion of significant differences in low frequency variant burden between populations. There are several potential explanations. First, since pathway bins are generally much larger than the other feature types, it is possible that large bins increase the false positive rate. Second, the same genes and regions can recur in multiple pathways. If the region has significant differences in low frequency variant burden, then each pathway or group containing that region will have a higher chance of having significant differences in low frequency variant burden. Following this logic, a pathway containing many genes has a higher chance of having at least one gene with extreme low frequency variant stratification. To compare only coding regions within a pathway, we filtered the pathway analysis to include only variants within exons. The proportions are reduced (shown in Figure S6) but still higher than the gene-exon proportions shown in Figure 3A.

### Evolutionary conserved regions (ECRs)

PhastCons output downloaded from UCSC Genome Browser was used to derive evolutionary conserved feature boundaries for primates, mammals, and more than 40 species of vertebrates. Figure S7 shows the 78 population comparisons for the ECR feature analysis. Of all of the feature analyses, ECR bins had the smallest proportion of significant bins. More ancestrally similar populations tended to have negligible low frequency burden differences in these conserved segments. For example, approximately 7% of the ECR region bins (vertebrate alignment) were significantly different between FIN (Finnish) and JPT (Japanese) individuals. However, the significant number of bins between the two ancestrally similar GBR (British) and CEU individuals was less than 1%.

### Regions of natural selection

To investigate regions of natural selection, we created a feature list using regions recently identified/confirmed by Grossman et al. with the Composite Multiple Signals algorithm on the 1000 Genomes Project data [18]. In addition, a publication by Barreiro et al. provided a list of specific genes with the strongest signatures of positive selection; i.e. genes that contained at least one nonsynonymous or 5′ UTR mutation with an F_ST_ value greater than 0.65 [19].

After lifting positions to build 37, there were only 368 regions from the regions identified by Grossman et al. The results are shown in Figure 6. The top plot corresponds to regions identified in African ancestry, the middle plot corresponds to regions identified in populations of Asian ancestry, and the bottom plot corresponds to regions identified specifically in populations of European ancestry. The trends in these three matrix plots are distinctly different from the trends shown in Figures 3–5. The blocks of comparisons within a continental group (shown in black boxes on each matrix plot) still have very little color, which means that the low frequency variant burden between populations within a continental group is very similar. The main difference is the gain of intensity outside of the continental groups. For example, in Figure 6B (regions identified in Asian populations), the European continental group and Spanish continental group mostly have proportions over 60% when compared to populations of Asian descent. In the same plot, the populations in the African group have proportions over 85% when compared to populations in the Asian group.

In general, we found regions considered to be under natural selection unlikely to have significant differences in low frequency burden between ancestrally similar populations, and very likely to have significant differences in regions considered to be under natural selection between ancestrally distant populations (see Figure 6). Additional analyses were performed using regions identified in other publications and can be found in Text S3, Table S2, and Table S3.

### Linkage disequilibrium in binned low frequency variants

**Figure 7:**
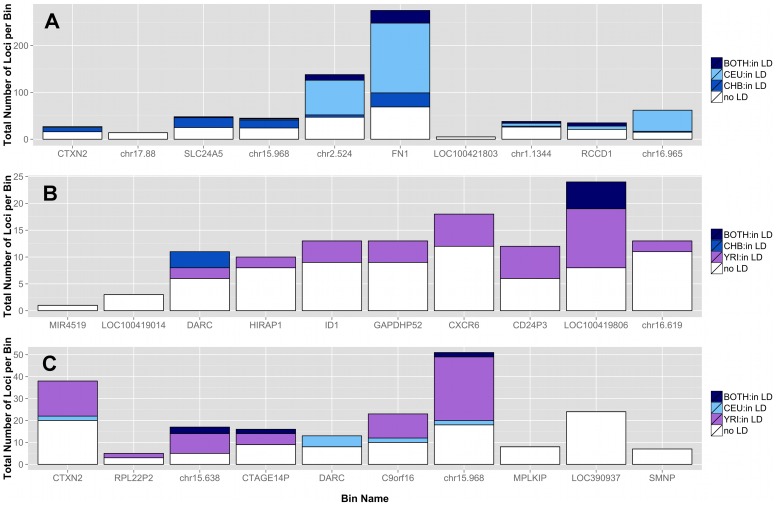
Proportion of loci in top bins in high LD with other variants in the same bin in A) CEU-CHB, B) CHB-YRI, and C) CEU-YRI population gene feature comparisons. Each bar represents a gene or intergenic bin. For a particular population comparison (A–C), the total height of the bar corresponds to the number of loci in that bin. The shades of blue and purple correspond to loci with r^2^ LD values greater than 0.3 for a specific population shown in the legend. The variant can be in LD in one population, the other population, or both (described in each legend). Almost all of the low frequency loci in LD had r^2^ values of approximately 0.5 or 1, corresponding to almost perfect LD. The white space corresponds to loci in the bin with LD values less than 0.3. The top bins are therefore, mostly composed of independent loci.

Although low frequency variants are commonly assumed as independent (in low linkage disequilibrium (LD) with other variants), there are rare haplotypes within related individuals and populations [20]. In Figure 7, three pairwise population comparisons are shown. We investigated the top 10 ranked bins from the CEU-CHB (A), CHB-YRI (B), and CEU-YRI (C) coding and noncoding analyses for presence of LD (r^2^>0.3) between two variants in the same bin. Figure 7 shows bins predominately filled with white-space indicating low to no pairwise LD between variants in those bins. In the top ten bins from these three analyses, rare haplotypes do not appear to be driving the significant differences seen in low frequency variant burden.

### Correlation between significance and bin size

Since the proportion of significant bins in the feature analyses is considerably higher for pathway bins than any other feature, we wanted to investigate the correlation between pathway p-value and bin size. We chose to assess the correlation between significance and several characteristics of the pathways using the pathway feature CEU-YRI population comparison. Figure S8 and Figure S9 show the correlations between six untransformed and transformed variables (with outliers removed), where each pairwise correlation is significant (p-value<0.05). A bin was considered an outlier if the number of loci in the bin was more than 2.5 standard deviations from the mean transformed loci value. The most interesting correlations were the nonlinear correlations between the loci/variants/genomic coverage and p-values. Figure S9B is a higher magnification of the highlighted correlation in Figure S9A, specifically; we plotted the correlation between −log_10_ p-values and log_10_ variants. The lowess smoothing function is shown in red, and the function appears to change slope twice. From x = 1 to x = 3, the slope is increasing with increasing number of variants. From x = 3 to x = 4, the slope is near 0. From x = 4 to x = 5, the slope is increasing with increasing numbers of variants. When the log_10_-transformed value of the number of variants is less than 3 or greater than 4, there appears to be a positive correlation between the number of variants in a bin and increasing significance of that bin. However, the data is not uniformly distributed and is sparse in those same areas. Therefore, the trends in the tails are most likely unreliable.

We created boxplots describing certain characteristics from each data source. Figure S10 shows that specific sources (i.e. KEGG) consistently have larger bin characteristics (number of loci, number of genes, coverage (kb), etc.) and also have much more significant bin p-values (Figure S10B). It appears that certain sources might inherently have more significant groups by nature of the information that source provides, or because of the size of groups found in the source.

From the matrix plots shown above, there is undoubtedly a functional component that influences the evolution of low frequency variants. However, from the correlations in the pathway analyses, it is also clear that larger bins can contain more stratification and thus more likely to have significance differences in low frequency burden. In more traditional case/control analyses, large bins are less likely to be significant because increasing binsize generally means more noise to mitigate the signal. However, in this study, when diverse populations are compared, larger bin sizes have more opportunity to capture population stratification.

Figure S11 shows the relative number of loci across tested features and varying interregion parameters. Boxplots in Figure S11A represent each feature tested in the population comparison. The small inset figure shows the magnitude of difference between the numbers of loci in pathways (peak) versus other feature types. The main plot in Figure S11A shows the same information, but is limited to 2000 loci. In general, ECRs/exonic regions/nonsynonymous gene variants/ORegAnno annotated regions/predicted deleterious gene variants/UTRs are very small bins. Pathway bins have a broader distribution, but in general are much larger. For comparison, we varied the size of intergenic regions (only noncoding regions) between 10 kb and 200 kb, those results are shown in Figure S11B. We also split the entire genome (including coding and noncoding regions) by various windows between 10 kb–200 kb. Figure S11C represents a genome “average,” and both Figure S11B and S11C can be used as comparison for feature tests. Figure S11B and S11C show increasing bin size as windows increase, the proportion of significant bins increase as window size increases as well (see Figure S12 and S13).

For example, Figure S13 shows matrix plots from whole-genome “average” analyses (A–G correspond to 10 kb, 25 kb, 50 kb, 75 kb, 100 kb, 150 kb, and 200 kb respectively). According to Figure S11A and S11C, exon bins from the original feature analysis are roughly comparable in size to 10 kb bins from the whole-genome “average” analysis. In the gene analysis results, approximately 43.9% of bins are significant after Bonferroni correction between CEU-YRI. Comparatively, the genome average between CEU-YRI for 10 kb bins is 57.64%. This supports the idea that coding regions are presumably more functional and perhaps more conserved than other regions in the genome of comparative size.

According to Figure S11A and S11C, pathway bins from the original feature analysis are roughly comparable in size to 150 kb bins from the whole-genome “average” analysis. In the pathway analysis results, approximately 81.28% of bins are significant after Bonferroni correction between CEU-YRI. Comparatively, the genome average between CEU-YRI for 150 kb bins is 86.09%. The gap between pathway bins and “average” genome stratification given similar size is much smaller for pathways than it is for exons. This particular pathway analysis includes introns (which typically have more variation than coding regions and larger bins are expected to collect more stratification. However, there are still fewer significant bins than expected on average.

## Discussion

### 1000 Genomes Project data

Since the reference genome is predominantly of European ancestry [21]–[23], populations with non-European ancestry generally have more variation with respect to the reference genome than those of European ancestry (see Table 1). Therefore, to interpret the results of this study, one might conclude that non-European populations have higher rates of sequencing error than European descent populations. However, in the most recent 1000 Genomes Project publication, the authors report an accuracy of individual genotype calls at heterozygous sites more than 99% for common SNVs and 95% for SNVs at a frequency of 0.5%. Furthermore, the authors found that variation in genotype accuracy was most related to sequencing depth and technical issues than population-level characteristics [11]. Therefore, neither the sequencing error nor the predominantly European reference genome adequately explains the trends seen in the genomic feature exploration (see Text S1, Table S1, Figures S1, S2).

Both sequence generation (technology and/or site) and population identity strongly contributes to underlying stratification in next-generation sequence data. After removing individuals with cryptic relatedness, 4 out of 13 Phase I populations were sequenced entirely using a single sequence technology (CHB, CHS, JPT, and TSI). The other 9 populations had between 3–18 individuals or ∼5%–57% of the population sequenced on technologies other than Illumina (ABI SOLID or LS454). Note: all three of the Asian populations (after removing individuals with cryptic relatedness) were sequenced only with Illumina technologies.

In our IBD analysis using variants with a minor allele frequency of 5% or greater and linkage disequilibrium r^2^< = 0.2, we identified and eliminated 75 individuals of various population backgrounds. In addition to the previously documented relatedness in 1000 Genomes Project [http://www.1000genomes.org/phase1-analysis-results-directory], we also found additional cryptic relatedness seen in other work [24], [25]. The differences are likely because we used continental groups (not a single population or the entire 1080 individuals) to identify cryptically related individuals and in our analysis that could include variants with fixed opposite frequencies and are overall common. This is infrequent in populations of the same continental group, but could be stratification introduced by different sequencing technologies.

### Genomic feature exploration

The major goal of this study was to investigate population stratification across multiple biological features. We created matrix plots to illustrate the *proportion* of significant bins in each comparison (shown in Figure 3, Figure 4, Figure 5, Figure 6, Figure S6, and Figure S7). Our results show an interesting trend between functional regions of the genome and variant tolerance. Mutations appear to be less tolerated in functional regions. Similarly, ECRs, which are known to be conserved among species, are also the features least likely to have variation burden differences between two populations. There is some debate about selection and functional significance in these conserved regions, it is unknown what factors have the largest effect on mutation rates [26], but it is possible that consistently low mutation rates in these features have generated conserved regions throughout evolution [27]. There are two potential explanations: 1) additional level of repair of DNA damage in transcriptional active regions by transcription coupled repair (TCR), 2) approximately 3% of the genome is subject to negative selection, however it is estimated that functionally dense regions contain up to 20% of the sites under selection [26], [27].

A number of the top results in each comparison have an interesting context, particularly in light of natural selection. Perhaps one of the most notable is *SLC24A5* (Ensembl ID:ENSG00000188467), which is one of the top ten results in 19 out of 78 populations comparisons in the gene feature analysis. European specific selective sweeps estimated in the last 20,000 years suggest that *SLC24A5* is key in skin pigmentation and Zebrafish with “golden” mutations exhibit melanosomal changes [28]–[30]. The presence of selection in particular populations due to environmental factors such as distance to the equator has led to the evolution and expansion of low frequency variants in some populations but not others. A second notable top result is *DARC* (Ensembl ID:ENSG00000213088), which encodes the Duffy antigen. The *DARC* gene bin was in the top ten results in 14 out of 78 population comparisons in the gene feature analysis. It has long been known that populations of African descent have increased diversity due to natural selection at this location, which prevents *Plasmodium vivax* infection.

The top result from the regulatory region analysis was a region on chromosome 20 (chr20:45395536–45396346) which was in the top ten bins in 24 out of 78 populations comparisons in the ORegAnno feature analysis. This region also overlaps ENCODE transcription factor binding sites in multiple cell lines, including: CTCF, POLR2A, NFYA, E2F1, FOS, and more. It was also annotated as an insulator in multiple cell lines in ENCODE Chromatin State Segmentation analyses using Hidden Markov Models [14], [31]. One last example, chr15.968, contains variants in the genome location chr15:48400199–48412256. This bin is one of the top ten bins in 17 out of 78 population comparisons in the intergenic analysis. The region covered by the chr15.968 bin is less than 1 kb upstream of *SCL24A5* on chromosome 15 and overlaps with several transcription factor-binding sites (including CTCF), regions thought to be weak enhancers, and regions thought to be insulators. According to Grossman et al., there are defined regions under natural selection before and after this region (chr15:45145764–45258860 and chr15:48539026–48633153), and all are very likely to participate in the transcriptional regulation of SLC24A5 [18].

The natural selection features require knowledge of three things for interpretation: 1) population A, 2) population B, and 3) the population where this signature was identified. When all three of these are within the same ancestral or continental group, we expect very few differences in low frequency burden. However, if population A is the same or similar to the population possessing the selection signature and population B is different, we expect significant differences in low frequency burden between population A and population B. In our results, we found that the vast majority of regions considered to be under natural selection had significant differences in low frequency burden between disparate ancestral populations, which support the theory of selection in these regions.

### Correlation between significance and bin size

In general, size of bins can influence the number of stratified variants contained and thus the significance of that bin. It is important to prove that this is because larger bins have a greater opportunity to “collect” variants that are stratified and not because of inflated type I error. We have tested type I error rates in bins between approximately 40 variants to over 100,000 variants, which covers all analyses presented in this paper, and found no correlation between bin size and Type I error rate (unpublished data). However, it should also be noted that while larger bins have more chances to collect stratified variants, there is also a larger capacity to collect neutral variants that contribute noise and decrease the signal.

Using CEU-YRI pathway burden analysis, we reviewed the correlation between pathway size and significance. The number of genes in pathways ranged from 1 to over 700 genes, with the average around 5 genes per group. Correlations for this data are shown in Figure S9. Not surprisingly, there were very linear and positive correlations between number of loci, number of variants, and genomic coverage. However, each of these had a nonlinear and somewhat complex relationship with the log-transformed p-value. This is highlighted in Figure S9B, which shows the relationship between the −log_10_ transformed p-value and the log_10_ transformed number of variants in the bin. The trend indicates that p-values are positively correlated (become more significant) with numbers of variants in a bin when the numbers of variants are relatively small or very large.

Two reasons could explain this correlation: 1) the false-positive rate is influenced by bin size (number of variants per bin), and 2) true signals from gene bins with burden differences perpetuate higher numbers of significant pathway bins. After extensive simulation testing (unpublished data) and recent publications in the literature, we believe the later is true [32], [33]. A single or small number of child bins (gene bins in this example), can drive parent bins (pathways in this example) to be significant even if no other child bin contains stratification.

The comparison in Figure S10 between group sources available in LOKI suggests KEGG, NetPATH, PharmGKB, and Reactome have consistently larger bins (higher number of loci, variants, and coverage). On average, these same four sources also tend to have bins with smaller p-values. Therefore, larger pathways are more likely to contain a gene with extreme low frequency variant stratification.

Population stratification is incredibly important in genomic analyses, particularly when low frequency variants are being studied. Expected stratification and potential bias is related to bin size and functional significance of region studied. Regions with more selective pressure often have fewer differences between populations than one would expect by chance. However, it is also important to consider the size of the region since population stratification tends to become more of a problem in large bins.

### Trends in the Asian continental group

The x-axis of each matrix plot (i.e. Figure 3) are oriented with African continental populations on the far right and the continental group with the highest proportion of significantly different low frequency variant bins on the far left. According to these matrix plots, Asian populations have more bins that are significantly different when compared to African populations than European/African population comparisons. Popular evolutionary theories suggest that the population that left Africa split before travelling East and West. One would expect low frequency burden differences (compared to African populations) to be very similar. However, populations from the Asian continental group tend to have more low frequency burden differences with African groups than European descent populations differences with African groups. There are at least three possible explanations; first, the Asian populations were the only continental group to be sequenced on the same technology, which could introduce a different bias when testing any of these populations with populations outside of Asian ancestry. While this is true of the 1,005 unrelated individuals, there were cryptically related individuals sequenced using SOLID technologies in all three of the Asian populations. The only population (including cryptically related individuals) to be sequenced exclusively on Illumina was TSI. When we examined the Asian populations and included the cryptically related individuals (and thus individuals sequenced with different technologies), the trend was the same. Asian populations are the most different from African populations with regard to low frequency variant burden. The second potential explanation is that Asian populations had considerable proportions of cryptic relatedness that had to be removed for our analysis, 49 of the 75 individuals removed were from Asian populations. Perhaps there was something unique about how those samples were collected. The third and most interesting explanation is a speculation that involves the journey for early Asian populations after leaving Africa. Travelling east was much different geographically than travelling west. For example, early Asian migrants would have traversed the Himalayan Mountains. The harsh travel could have induced bottlenecks and other evolutionary mechanisms that would uniquely change the genetic architecture, specifically the architecture of low frequency variation. The course of travel for European descent populations was very different; they were exposed to unique challenges and climates. As each continental group diverged from Africa, their separate paths could explain why the difference in burden exists (EAS/AFR and EUR/AFR).

### Conclusion

As we continue in pursuit of genetic etiology explaining heritability in common, complex disease, it is important to consider multiple types of genomic data, specifically variation beyond common variants. Low frequency variants are more frequent in the genome than common variants and are likely to have significant functional impact on human health. However, as we look forward to many successes in next-generation data analysis, it has become increasingly clear that we can't apply the same methods and corrections to low-frequency variants as we did in GWAS. Since low frequency variants are often recent mutations, they are specific to continental ancestry groups. This provides two important conclusions. First, potentially functional low-frequency variants are likely not the same across distantly related individuals. Second, low frequency population substructure leads to substantial differentiation and cannot be ignored [11].

Until relatively recently, we have not focused on the challenges presented by low frequency population stratification. Current methods used for GWAS to correct for ancestry are not likely adequate for low frequency stratification [34], [35]. Therefore, it is imperative that researchers are aware of potential pitfalls stratification can introduce to low frequency genomic analyses.

In summary, we were able to expose the magnitude of low frequency population stratification between all populations available in 1000 Genomes Project Phase I release across multiple interesting biological features. The magnitude of low frequency stratification appeared to be dependent on the functional location of the variation and the genomic size of the pertinent features. For example, there were fewer differences in low frequency burden in coding regions than intergenic regions. We found features with less variant tolerance and possibly more evolutionary constraint to have fewer differences in low frequency variant burden between different populations, i.e. significant low frequency bins seemed to be consistent with mutation theory. In addition, larger features were more likely to contain stratified variants and thus be significantly different between two populations. Low levels of stratification existed even between populations of the same continental group. The results of this study serve as a warning to researchers whom wish to use population control groups such as 1000 Genomes Project or shared control sets, unmatched case and control groups can contribute significantly to type I error rates. Future studies should focus on methods to accurately control for low frequency population stratification.

## Methods

### BioBin software

BioBin is a standalone command line application written in C++ that uses a prebuilt LOKI database described below (software paper in preparation). Source distributions are available for Mac and Linux operating systems and require minimal prerequisites to compile. Included in the distribution are tools that allow the user to create and update the LOKI database by downloading information directly from source websites. The computational requirements for BioBin are quite modest; for example, during testing, a whole-genome analysis including 185 individuals took just over two hours using a single core on a cluster (Intel Xeon X5675 3.06 GHz processor). However, because the vast amount of data included in the analysis must be stored in memory, the requirements for memory usage can be high; the aforementioned whole-genome analysis required approximately 13 GB of memory to complete. Even with large datasets, BioBin can be run quickly without access to specialized computer hardware or a computing cluster. The number of low frequency variants is the primary driver of memory usage [36]. BioBin is open-source and publicly available on the Ritchie lab website (http://ritchielab.psu.edu/ritchielab/software/).

### Library of Knowledge Integration (LOKI) database

Harnessing prior biological knowledge is a powerful way to inform collapsing feature boundaries. BioBin relies on the Library of Knowledge Integration (LOKI) for database integration and boundary definitions. LOKI contains resources such as: the National Center for Biotechnology (NCBI) dbSNP and gene Entrez database information (downloaded dbSNP b137: Dec 21 2012, Entrez: Feb 1 2013) [37], Kyoto Encyclopedia of Genes and Genomes (KEGG, downloaded Dec 21 2012, Release 64) [38], Reactome (downloaded Dec 12 2012) [39], Gene Ontology (GO, downloaded Feb 1 2013) [40], Protein families database (Pfam, downloaded Dec 1 2011) [41], NetPath - signal transduction pathways (downloaded Sept 3 2011) [42], Molecular INTeraction database (MINT, downloaded Oct 29 2012) [43], Biological General Repository for Interaction Datasets (BioGrid, downloaded Feb 1 2013, version 3.2.97) [44], Pharmacogenomics Knowledge Base (PharmGKB, downloaded Jan 6 2013) [45], Open Regulatory Annotation Database (ORegAnno, downloaded Jan 10 2011) [46], and evolutionary conserved regions from UCSC Genome Browser (downloaded Nov 10 2009) [14].

LOKI provides a standardized interface and terminology to disparate sources each containing individual means of representing data. The three main concepts used in LOKI are *positions*, *regions* and *groups*. The term *position* refers to single nucleotide polymorphisms (SNPs), single nucleotide variants (SNVs) or low frequency variants. The definition of *region* has a broader scope. Any genomic segment with a start and stop position can be defined as a region, including genes, copy number variants (CNVs), insertions and deletions, and evolutionary conserved regions (ECRs). *Sources* are databases (such as those listed above) that contain *groups* of interconnected information, thus organizing the data in a standardized manner.

LOKI is implemented in SQLite, a relational database management system, which does not require a dedicated database server. The user must download and run installer scripts (python) and allow for 10–12 GB of data to be downloaded directly from the various sources. The updater script will automatically process and combine this information into a single database file (∼6.7 GB range). A system running LOKI should have at least 50 GB of disk storage available. The script to build LOKI is open source and publicly available on the Ritchie lab website (http://ritchielab.psu.edu/ritchielab/software/). Users can customize their LOKI database by including or excluding sources, including additional sources, and updating source information as frequently as they like [36].

### Binning approach

**Figure 8:**
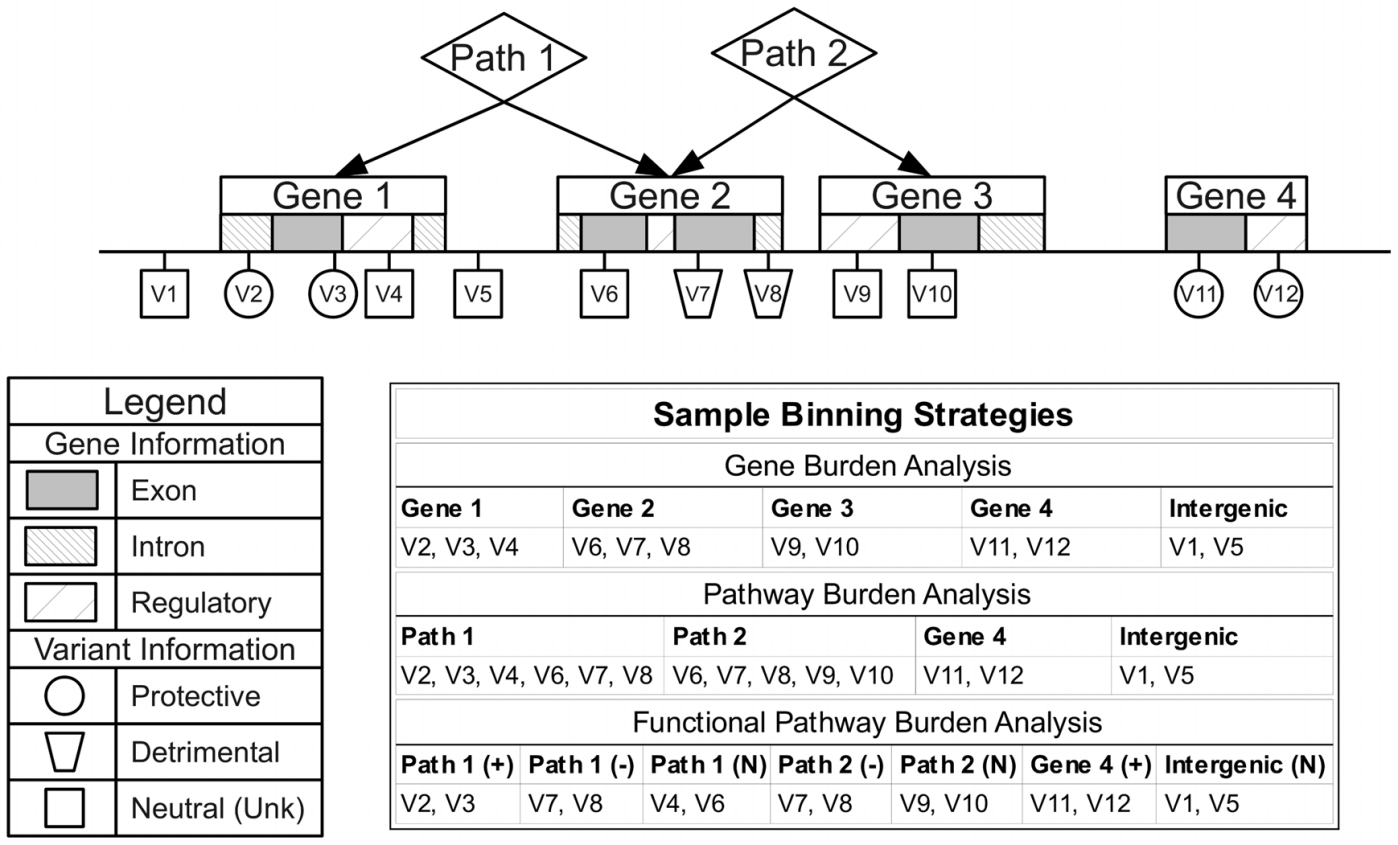
Alternate binning strategies using biological knowledge and functional or role annotations. Three example binning strategies: gene burden analysis, pathway burden analysis, and functional pathway burden analysis using four genes, two pathways, and variant functional prediction information.

We chose NCBI dbSNP and NCBI Entrez Gene as our primary sources of position and regional information due the quality and reliability of the data, clearly defined database schema, and because they contain gene IDs that map to the majority of group sources in LOKI. Gene boundary definitions were derived from NCBI Entrez. Pathway/group bins, regulatory regions, and evolutionary conserved regions were created using sources available in LOKI (sources detailed in Software section). Some sources explicitly provide lists of genes in pathways; others provide groups of genes, which share a biological connection (i.e. protein-protein interactions). For the purposes of this study, any bin created by multiple regions/genes will be analyzed in the “Pathway-Groups” feature analysis. External custom input files were generated using boundaries of annotated exon regions from UCSC to bin exon and intron specific variants. For example, if Gene A has three exons and two introns, only two bins would be created: GeneA-exons and GeneA-introns. GeneA-exons would contain all variants that fell within any of the three Gene A exon boundaries. External custom feature files were also generated for regions under natural selection by combining regions provided by previously published work [18], [19]. Example binning strategies can be seen in Figure 8. Using hierarchical biological relationships and optional functional or role information, BioBin can create many combinations of variants to bin. Custom feature files and additional binning details are explained in Text S4, Table S4, and Table S5.

### Statistical analysis

BioBin is a bioinformatics tool used to create new feature sets that can then be analyzed in subsequent statistical analyses. Statistical tests used with BioBin can be chosen according to the hypothesis being tested, the question of interest, or the type of data being tested [36]. Unless otherwise noted, the results presented herein were calculated using a Wilcoxon 2-sample rank sum test implemented and graphed in the R statistical package [47], [48]. P-values presented have been corrected using a standard Bonferroni correction, adjusting for the number of bins created and tested in a given analysis. Simulations confirming the power and validity of using the Wilcoxon 2-sample rank sum test are described in Text S5 and Table S6.

### Low frequency variant burden analysis in 1000 Genomes Project data

To investigate population stratification using BioBin, we analyzed the 1000 Genomes Project Phase I data. The 1000 Genomes Project was started in 2008 with the mission to provide deep characterization of variation in the human genome. As of October 2011, the sequencing project included whole-genome sequence data for 1080 individuals, and aimed to sequence 2,500 individuals by its completion [49]. We removed 75 cryptically related individuals and conducted a pairwise comparison of low frequency variant burden differences between all 13 ancestry groups included in the phase I release of the 1000 Genomes Project (October 2011 release). Table 1 provides the total number of variants (common and low frequency) and individuals included in Phase I VCF files of 1000 Genomes Project data for 1080 individuals in all 13 populations.

### Investigation of allele sharing

In any genetic study, and especially in consideration of low frequency variants, it is important to evaluate sample relatedness. We combined populations by continental ancestry (i.e. AFR continental group includes ASW, LWK, YRI) and evaluated sample relatedness between and within the general ancestry groups using identity-by-state (IBS) and identity-by-descent (IBD). Pairwise IBS represents the number of shared alleles at a specific locus between two individuals. IBS can be observed as 0, 1, or 2 depending on how many alleles are in common between the pair. If the shared alleles are inherited from a recent common ancestor, they are also considered IBD. Pairwise IBS calculations for low-frequency variants approximate IBD since the variants are likely to be recent and the chance of being identical because of recurrence is rare [50].

We used plink and plink-seq to estimate pairwise IBS and IBD for individuals of the same general ancestry group (http://atgu.mgh.harvard.edu/plinkseq/, http://pngu.mgh.harvard.edu/~purcell/plink/) [51]. For common variants, we created an independent subset of SNVs with a minor allele frequency greater than 5% and r^2^ values less than 0.2 to calculate pairwise IBD between individuals. For example, for the populations of African descent (LWK, ASW, and YRI) we grouped all of the individuals from these three populations and calculated the IBD. We removed maximally connected or related individuals in a parsimonious and iterative manner and repeated the IBD analysis until the maximum pairwise pi_hat score was less than or equal to 0.3. After repeating this analysis in each continental group, 75 individuals were dropped from BioBin analyses based on our threshold for cryptic relatedness. We also evaluated allele sharing within and between major ancestral groups using plink-seq to calculate IBS for low frequency variants and common variants (threshold 0.03 MAF and 0.25 MAF, respectively). Even though we estimated IBD in common variants (described above), we calculated the IBS in low frequency and common variants separately to ensure the results were consistent. Using the ratio of shared alleles divided by the total number of genotyped alleles between two individuals, we evaluated excess sharing of low frequency variants (MAF<0.03) compared to excess sharing of common variants (MAF>0.25).

### Genomic feature exploration

Feature selection in BioBin is a clear innovation over other available collapsing methods. Knowledge of biological features, such as genes and pathways, are available through LOKI for binning. In this analysis, we used the feature options of BioBin to investigate a variety of biologically relevant bins for differences in low frequency variant burden across 13 populations. We implemented a minimum bin size of two variants, inter-region bin size of 50 kb, and set the MAF binning threshold to 0.03. We chose a 3% MAF binning threshold to focus our analysis on rare and near rare variation that differs between population groups. Additional details concerning binning parameters can be found in the Text S4. We binned genes (introns, exons, nonsynonymous variants, and predicted deleterious variants), intergenic regions, pathways, pathway-exons, regulatory regions, evolutionary conserved regions, and regions thought to be under natural selection.

Natural selection can alter genomic variation in features, particularly in regions with some impact on protein function (regulatory regions, coding regions). Positive selection on a specific variant allows the advantageous variant to sweep through a population, which can lead to an excess of common variants. Alternatively, weak negative selection or purifying selection can result in selective removal of deleterious alleles. This can decrease variation around the locus under selection and lead to an excess of rare or low frequency variation [52]. Commonly, evidence of natural selection is found only in one ancestral group, which is consistent with the idea that these selection events postdate population separation [53]. Because of this differentiation among populations, we were interested in using regions identified as being under selective pressures as features in a BioBin analysis. Table 2 shows the analysis plan, features tested, sources used, and the mean number of bins generated across all pairwise comparisons.

**Table 2:** Binning analysis overview.

ANALYSIS	FEATURE	SOURCES	AVG BIN TOTAL
A	Genes-Exons (NS/DEL)	NCBI Entrez, UCSC roles	80786
	Genes-Introns	NCBI Entrez, UCSC roles	
	Genes-Unknown	NCBI Entrez	
	Intergenic (50 kb)	-	
B	Pathways/Groups	PFAM, KEGG, NetPath, PharmGKB, MINT, GO, dbSNP, Entrez, Reactome	178497
C	Natural Selection	Grossman	368
D	ORegAnno	UCSC-ORegAnno	11293
E	ECR-vertebrates	UCSC-PhastCons	319269
	ECR-placental mammals		
	ECR-primates		

Analyses performed for each population comparison; including, features tested, contributing sources, and total of bins generated for each binning analysis.

After evaluating the population comparisons for the features described in Table 2, we investigated the linkage disequilibrium (LD) in 10 top-ranked bins for three population comparisons, CEU-CHB, CHB-YRI, CEU-YRI. We calculated the LD between binned variants and determined the number of variants inside of a bin in LD with an r^2^> = 0.3. We also evaluated the correlation between pathway significance and bin size. We took all of the pathways in the YRI/CEU analysis and compiled the following information for each pathway bin; total genomic coverage, number of genes, number of independent genes, number of loci, number of variants, and BioBin p-value. Because the majority of pathways or groups are not very large, the data was heavily skewed (see Figure S8). We performed a log_10_ transformation on all six variables: number of genes in the pathway or group, number of unique genes (not present in any other pathway or group), number of loci in the pathway bin, number of variants in the pathway bin, genomic coverage of the pathway bin, and the BioBin reported Bonferroni adjusted p-value. Because of the skewness, we removed any pathway bins that had transformed loci values outside of 2.5 standard deviations of the log-transformed loci mean.

## Supporting Information

Figure S1 Investigating differential bias in 1000 Genomes Project data using principal components analysis. Each plot shows the first two principal components calculated from each continental group colored by population identity. Additionally, the shapes of the points indicate technology used, with circles representing ABI SOLiD and plusses representing Illumina platforms The labels correspond to populations from four continental groups: (A) EUR continental group, (B) EAS continental group, (C) AMR continental group, and (D) AFR continental group. Since the global variation is caused primarily by sequence technology, and very few populations are actually sequenced on a single technology, sequence technology likely contributes little bias to the trends seen in our results.
(TIF)

Figure S2 Sample analysis with two methods of correcting for technology effects. A) A sample analysis using gene bins with a MAF binning threshold of 5% tested with Firth logistic regression. B) The same analysis with the use of principal component covariates with the population stratification effects removed. In this analysis, the principal component covariates are able to correctly predict the technology with 95% accuracy on average. C) The same analysis using the sequencing technology itself as covariates in the regression. In both methods of correction for technology effects (B,C), we show no substantial influence of technology effects on the population stratification.
(TIF)

Figure S3 IBD estimates using variants with MAF>10% within and between ancestral groups. There are two frames for each ancestral group. The labels correspond to populations from four continental groups: (A/B) AFR continental group, (C/D) EUR continental group, (E/F) EAS continental group, and (G/H) AMR continental group. The left frame from each continental group corresponds to the IBD estimate within each population (A, C, E, G). The right frame from each continental group corresponds to the IBD estimate between populations within the ancestral group (B, D, F, H).
(TIF)

Figure S4 Pairwise IBS calculations for low frequency variants (MAF<3%) within continental groups. Plots (A–D) show the IBS calculations within continental groups for all 1080 individuals. The plots to the right (E–H) shows the IBS calculations within continental groups for 1005 individuals (cryptically related individuals removed). Each dot represents a pair of individuals; the colors correspond to population comparisons. Points with the higher mean IBS indicate increased sharing.
(TIF)

Figure S5 Pairwise IBS calculations for common variants (MAF>25%) within continental groups (A–D). Plots (A–D) show the IBS calculations within continental groups for all 1080 individuals. The plots to the right (E–H) shows the IBS calculations within continental groups for 1005 individuals (cryptically related individuals removed). Each dot represents a pair of individuals; the colors correspond to population comparisons. Points with the higher mean IBS indicate increased sharing.
(TIF)

Figure S6 Proportion of significantly different bins for the pathway-exon feature analysis. The numbers in each block and the color intensity [0,1] indicate the proportion of significant bins for the 1000 Genomes populations on each axis. In general, the x-axis is organized with African descent populations on the far right and increasing differentiation with regard to low frequency burden towards the left (i.e. populations of Asian descent have the highest proportion of significant bins compared to African descent groups). The overall proportion of significant bins is much less in this pathway-exon analysis than the pathway analysis shown in Figure 5b.
(TIF)

Figure S7 Proportion of significantly different bins in evolutionary conserved region feature analysis (A) conserved with primates, (B) conserved with mammals, and (C) conserved with vertebrates. The numbers in each block and the color density indicate the proportion of significant bins for the 1000 Genomes populations on each axis. For example, in the ECR: vertebrate matrix, 16.38% of the ECR bins have significant differences in low frequency burden between YRI and CHS populations. In general, the x-axis is organized with African descent populations on the far right and increasing differentiation with regard to low frequency burden towards the left (i.e. populations of Asian descent have the highest proportion of significant bins compared to African descent groups).
(TIF)

Figure S8 Investigation of pathway significant correlation with binsize using untransformed pathway variables. Correlation scatterplot matrix for six untransformed variables: the number of genes in a pathway (n_genes), the number of unique genes in the pathway (n_uniq), the number of loci in the pathway bin (loci), the number of variants in the pathway bin (variants), the genomic coverage of pathway (coverage_kb), and the bin p-value (p-val). Bins considered outliers were removed before generating the correlations (http://stat.ethz.ch/R-manual/R-patched/library/graphics/html/pairs.html). The variables are right skewed and require transformation.
(TIF)

Figure S9 Investigation of pathway significant correlation with binsize using log_10_ transformed pathway variables. A) Correlation scatterplot matrix for six log_10_ transformed variables: the number of genes in a pathway (n_genes), the number of unique genes in the pathway (n_uniq), the number of loci in the pathway bin (loci), the number of variants in the pathway bin (variants), the genomic coverage of pathway (coverage_kb), and the bin p-value (p-val), B) higher magnification of the correlation highlighted in Figure S8A, but instead of the +log10 transform of p-values, it is showing the the −log_10_ transformed p-values and log_10_ transformed variants with a loess smoothing function (red line) and 95% confidence intervals (gray shading). Bins considered outliers were removed before generating the correlations. The number of loci, number of variants, and size of genomic region were significantly and linearly correlated with each other (correlation coefficients >0.95). On the x-axis, the slope from x = 1 to x = 3 is relatively linear and the −log_10_ p-value increases with increasing number of variants (p-value becomes more significant). From x = 3 to x = 4, the slope is near 0. From x = 4 to x = 5, the slope appears nonlinear and with a larger slope than the left slope, indicating again most significant p-values with higher numbers of variants in a bin. Although these are transformed values, the p-values are not perfectly uniform. Therefore, the tails are possibly unreliable (http://stat.ethz.ch/R-manual/R-patched/library/graphics/html/pairs.html).
(TIF)

Figure S10 Pathway characteristics presented by LOKI source. Different pathway characteristics presented in box plots: A) The y-axis shows the log_10_ frequency of each source statistic for the number of genes (Num. Genes), the number of loci (Num. Loci), the number of variants (Num. Variants), and the coverage in kb, B) The distribution of p-values for the various knowledge sources. On average, the same four sources listed above also tend to have bins with smaller p-values. Each boxplot and color corresponds to the biological knowledge sources listed in the legend. KEGG, NetPATH, PharmGKB, and Reactome show consistently larger bins (higher number of loci, variants, and coverage).
(TIF)

Figure S11 Bin size distribution (loci per bin) across features tested in population comparison and various intergenic and whole-genome bins. A) bin size for each feature across all population comparisons, B) intergenic bins with variable window sizes (excluding any/all coding regions), C) whole-genome bins with variable window sizes. Each plot has a small inset with a complete picture of the loci distribution. The larger plots are zoomed to Y[0∶2000] in order to compare between features and bin sizes.
(TIF)

Figure S12 Proportion of significantly different bins for the intergenic feature analysis with variable window sizes (excluding any coding regions). A) 10 kb window, B) 25 kb window, C) 50 kb window, D) 75 kb window, E) 100 kb window, F) 150 kb window, G) 200 kb window. The numbers in each block and the color intensity [0,1] indicate the proportion of significant bins for the 1000 Genomes populations on each axis. In general, the x-axis is organized with African descent populations on the far right and increasing differentiation with regard to low frequency burden towards the left (i.e. populations of Asian descent have the highest proportion of significant bins compared to African descent groups).
(TIF)

Figure S13 Proportion of significantly different bins for the whole genome feature analysis with variable window sizes. A) 10 kb window, B) 25 kb window, C) 50 kb window, D) 75 kb window, E) 100 kb window, F) 150 kb window, G) 200 kb window. The numbers in each block and the color intensity [0,1] indicate the proportion of significant bins for the 1000 Genomes populations on each axis. In general, the x-axis is organized with African descent populations on the far right and increasing differentiation with regard to low frequency burden towards the left (i.e. populations of Asian descent have the highest proportion of significant bins compared to African descent groups).
(TIF)

Table S1 Phase I 1000 Genomes Project sequence technology data characteristics. 1000 Genomes Project Phase I populations (1080 individuals, 13 populations) and number of individuals from each population sequenced on ABI solid, Illumina, LS454 or Illumina and LS454.
(PDF)

Table S2 Reviewing regions of interest found in Barreiro study. Genes identified in Barreiro study with an F_ST_ value >0.65 that were also found in the regions identified by Pritchard (J.P.), Stoneking (M.S.), and Sabeti (P.S.) [18], [54], [55].
(PDF)

Table S3 Specific genes of interest with known allele frequency differences between ancestral populations.
(PDF)

Table S4 Example of BioBin parameter options, rare-case-control (RCC) and overall-major-allele (OMA). Table S4 contains iterations of major/minor allele selection and variant binning using new parameters rare-case-control (RCC) and overall-major-allele (OMA). Using both options is necessary to make the results independent of control group selection.
(PDF)

Table S5 Excerpt of custom region file containing regions with signatures of natural selection.
(PDF)

Table S6 Low frequency simulation using SimRare to assess Type I error and power.
(PDF)

Text S1 Differential bias in 1000 Genomes Project data.
(DOCX)

Text S2 Investigation of allele sharing in 1080 individuals.
(DOCX)

Text S3 Additional results from regions of natural selection analysis.
(DOCX)

Text S4 Additional binning algorithm details.
(DOCX)

Text S5 Data simulation strategy.
(DOCX)
