# Supplementary figures and images for: Low Frequency Variants, Collapsed Based on Biological Knowledge, Uncover Complexity of Population Stratification in 1000 Genomes Project Data
Source: PLoS Genet. 2013 Dec 26;9(12):e1003959. doi: 10.1371/journal.pgen.1003959 (PMC3873241; doi:10.1371/journal.pgen.1003959)

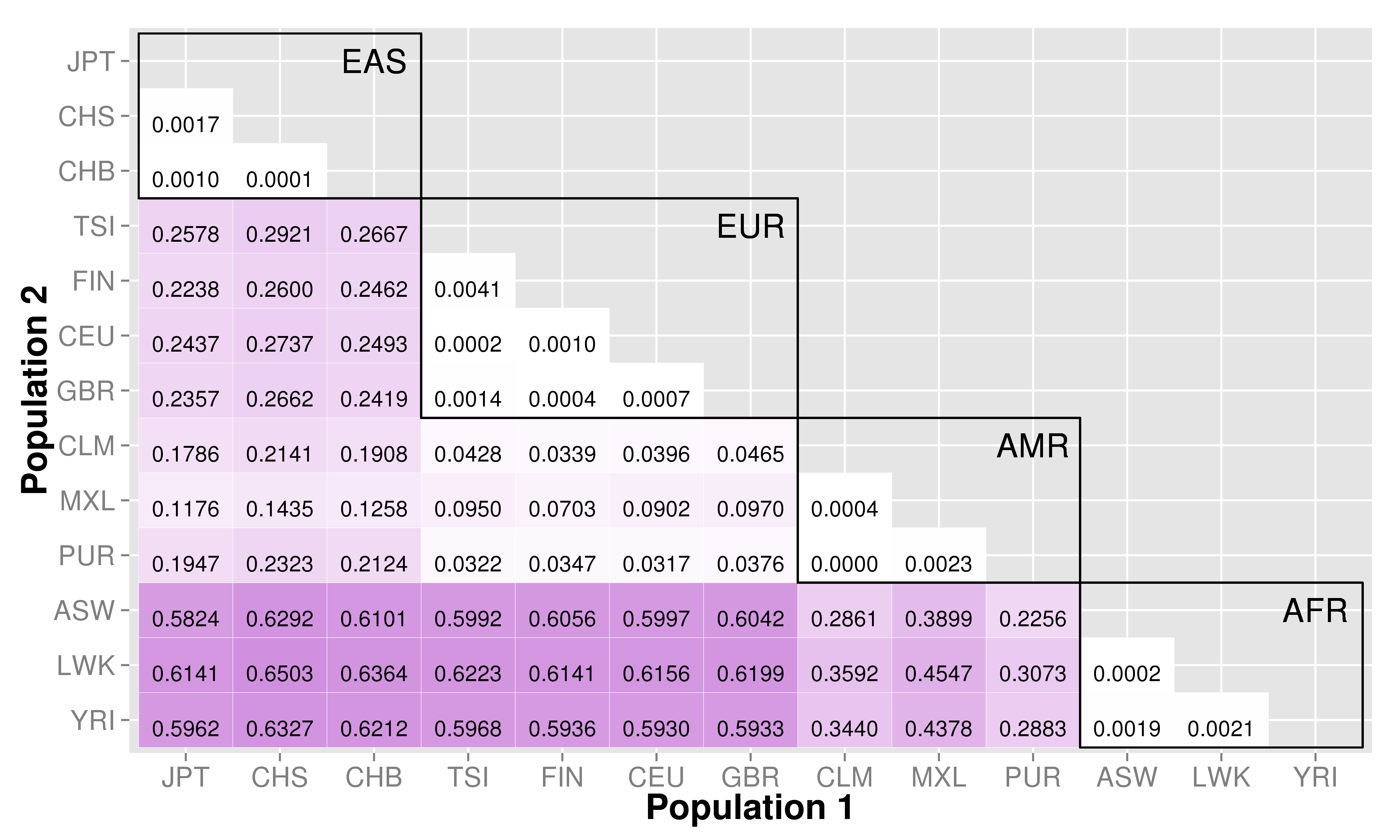

Supplement: Figure S6 — Proportion of significantly different bins for the pathway-exon feature analysis. The numbers in each block and the color intensity [0,1] indicate the proportion of significant bins for the 1000 Genomes populations on each axis. In general, the x-axis is organized with African descent populations on the far right and increasing differentiation with regard to low frequency burden towards the left (i.e. populations of Asian descent have the highest proportion of significant bins compared to African descent groups). The overall proportion of significant bins is much less in this pathway-exon analysis than the pathway analysis shown in Figure 5b. (TIF) [file pgen.1003959.s006.tif]

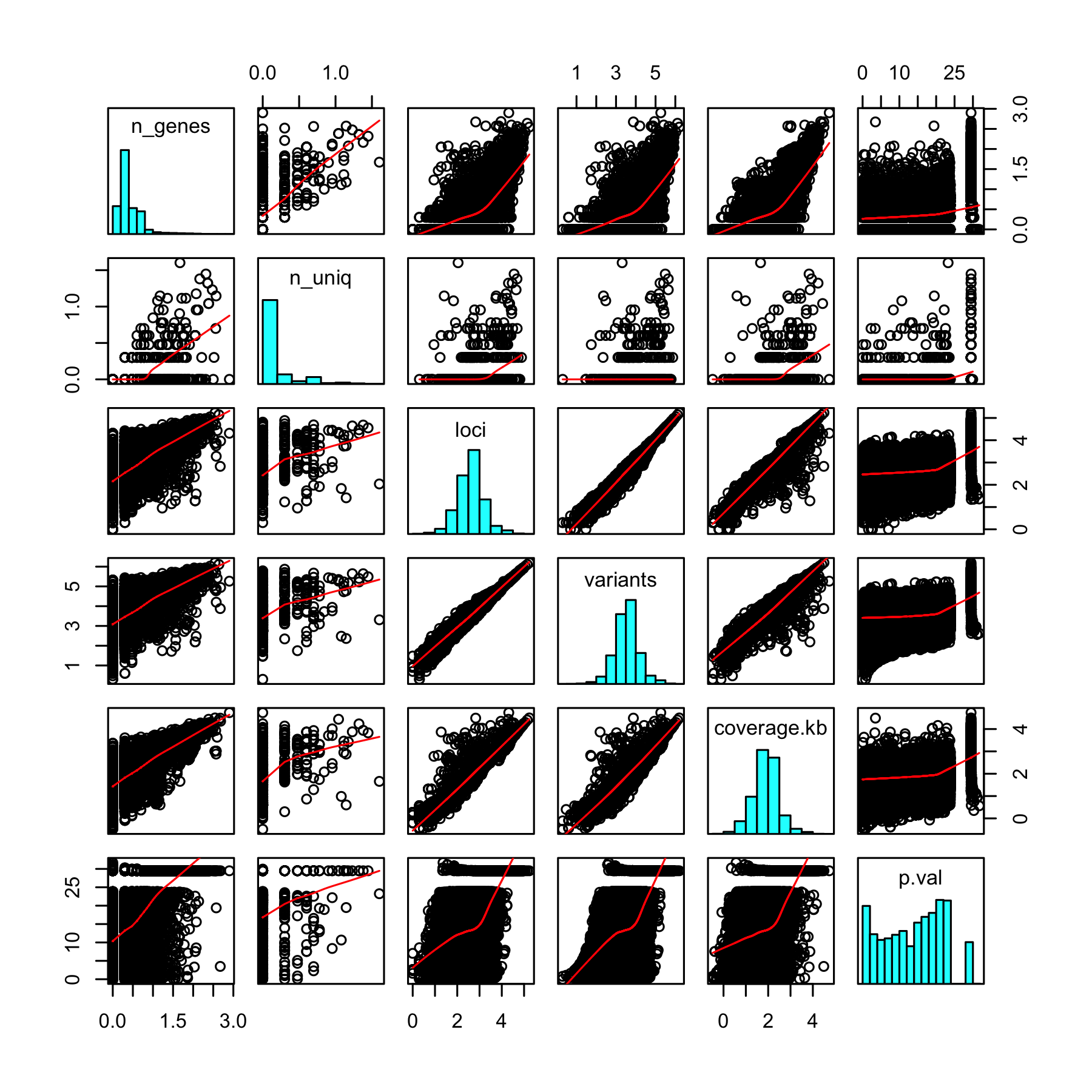

Supplement: Figure S8 — Investigation of pathway significant correlation with binsize using untransformed pathway variables. Correlation scatterplot matrix for six untransformed variables: the number of genes in a pathway (n_genes), the number of unique genes in the pathway (n_uniq), the number of loci in the pathway bin (loci), the number of variants in the pathway bin (variants), the genomic coverage of pathway (coverage_kb), and the bin p-value (p-val). Bins considered outliers were removed before generating the correlations (http://stat.ethz.ch/R-manual/R-patched/library/graphics/html/pairs.html). The variables are right skewed and require transformation. (TIF) [file pgen.1003959.s008.tif]

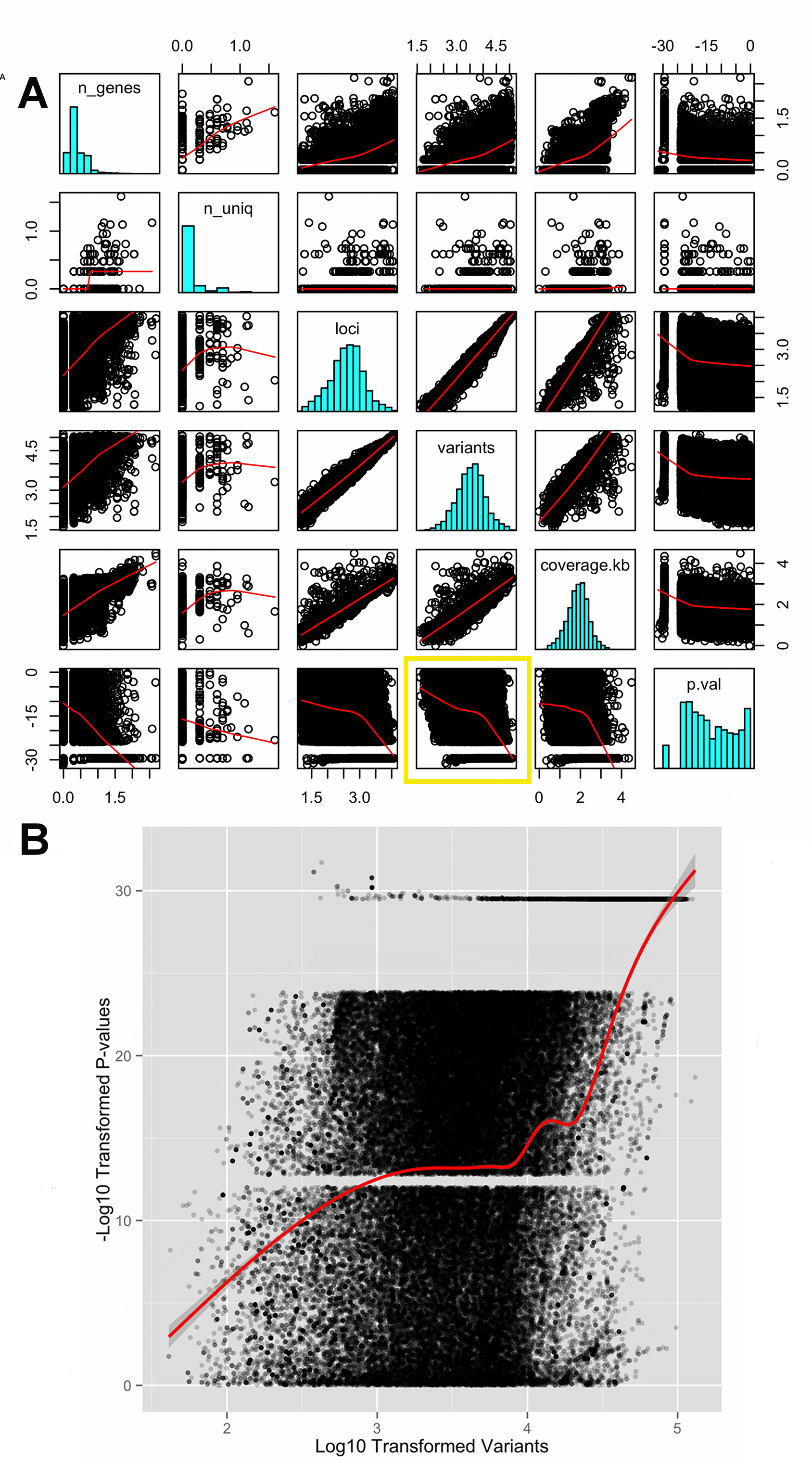

Supplement: Figure S9 — Investigation of pathway significant correlation with binsize using log10 transformed pathway variables. A) Correlation scatterplot matrix for six log10 transformed variables: the number of genes in a pathway (n_genes), the number of unique genes in the pathway (n_uniq), the number of loci in the pathway bin (loci), the number of variants in the pathway bin (variants), the genomic coverage of pathway (coverage_kb), and the bin p-value (p-val), B) higher magnification of the correlation highlighted in Figure S8A, but instead of the +log10 transform of p-values, it is showing the the −log10 transformed p-values and log10 transformed variants with a loess smoothing function (red line) and 95% confidence intervals (gray shading). Bins considered outliers were removed before generating the correlations. The number of loci, number of variants, and size of genomic region were significantly and linearly correlated with each other (correlation coefficients >0.95). On the x-axis, the slope from x = 1 to x = 3 is relatively linear and the −log10 p-value increases with increasing number of variants (p-value becomes more significant). From x = 3 to x = 4, the slope is near 0. From x = 4 to x = 5, the slope appears nonlinear and with a larger slope than the left slope, indicating again most significant p-values with higher numbers of variants in a bin. Although these are transformed values, the p-values are not perfectly uniform. Therefore, the tails are possibly unreliable (http://stat.ethz.ch/R-manual/R-patched/library/graphics/html/pairs.html). (TIF) [file pgen.1003959.s009.tif]

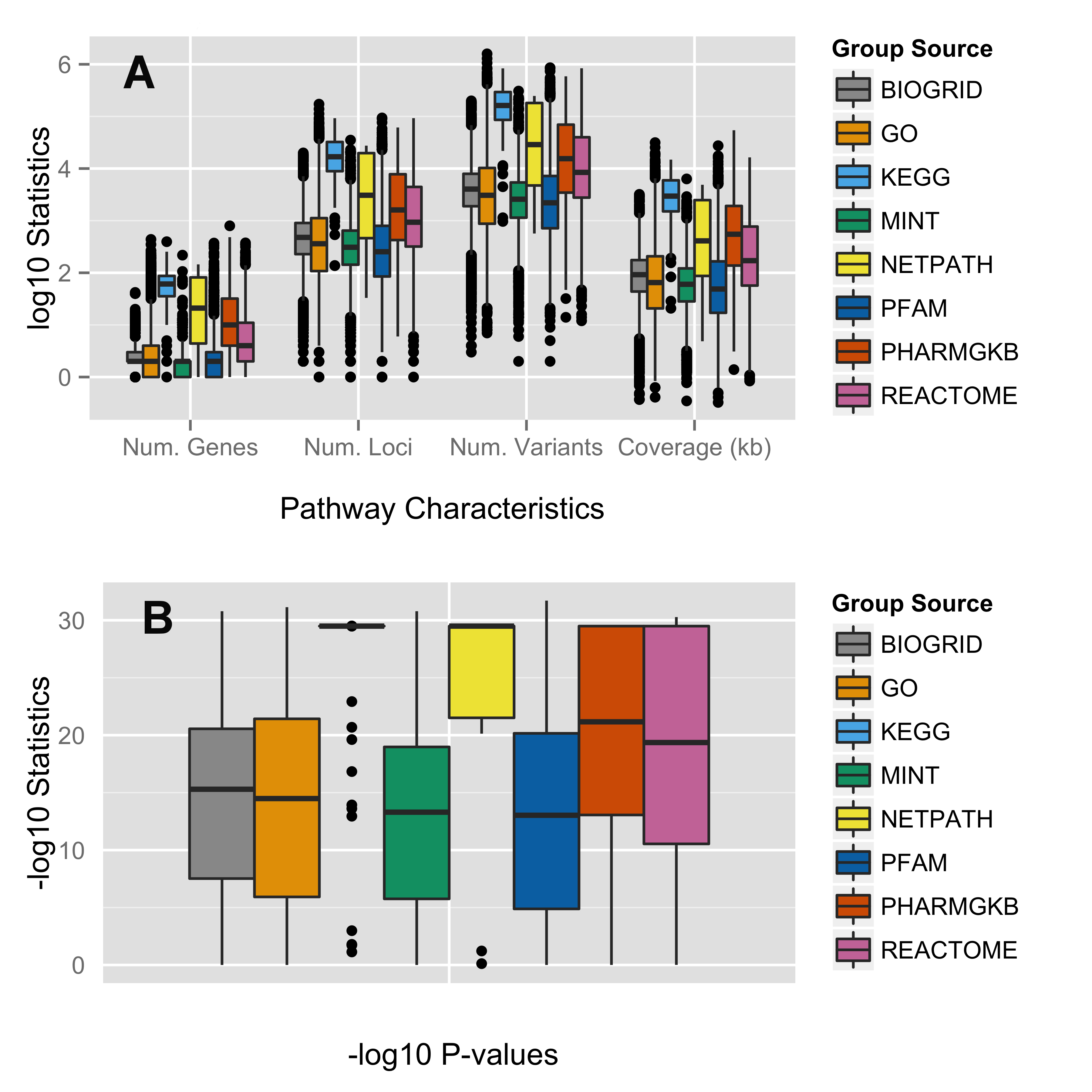

Supplement: Figure S10 — Pathway characteristics presented by LOKI source. Different pathway characteristics presented in box plots: A) The y-axis shows the log10 frequency of each source statistic for the number of genes (Num. Genes), the number of loci (Num. Loci), the number of variants (Num. Variants), and the coverage in kb, B) The distribution of p-values for the various knowledge sources. On average, the same four sources listed above also tend to have bins with smaller p-values. Each boxplot and color corresponds to the biological knowledge sources listed in the legend. KEGG, NetPATH, PharmGKB, and Reactome show consistently larger bins (higher number of loci, variants, and coverage). (TIF) [file pgen.1003959.s010.tif]
