# Supplementary material for: Low Frequency Variants, Collapsed Based on Biological Knowledge, Uncover Complexity of Population Stratification in 1000 Genomes Project Data
Source: PLoS Genet. 2013 Dec 26;9(12):e1003959. doi: 10.1371/journal.pgen.1003959 (PMC3873241; doi:10.1371/journal.pgen.1003959)
Supplement: Table S1 — Phase I 1000 Genomes Project sequence technology data characteristics. 1000 Genomes Project Phase I populations (1080 individuals, 13 populations) and number of individuals from each population sequenced on ABI solid, Illumina, LS454 or Illumina and LS454. (PDF) [file pgen.1003959.s014.pdf]

Continental Group	Population	ABI_SOLID	ILLUMINA	ILLUMINA,LS454	LS454
African descent (AFR)	ASW	11	50	0	0
	LWK	14	83	0	0
	YRI	12	76	0	0
Asian descent (EAS)	CHB	16	81	0	0
	CHS	8	92	0	0
	JPT	11	78	0	0
European descent (EUR)	CEU	0	72	9	6
	FIN	18	75	0	0
	GBR	19	70	0	0
	TSI	0	98	0	0
Spanish/Mexican descent (AMR)	CLM	10	50	0	0
	MXL	12	54	0	0
	PUR	3	52	0	0
