# Supplementary material for: Low Frequency Variants, Collapsed Based on Biological Knowledge, Uncover Complexity of Population Stratification in 1000 Genomes Project Data
Source: PLoS Genet. 2013 Dec 26;9(12):e1003959. doi: 10.1371/journal.pgen.1003959 (PMC3873241; doi:10.1371/journal.pgen.1003959)
Supplement: Table S2 — Reviewing regions of interest found in Barreiro study. Genes identified in Barreiro study with an FST value >0.65 that were also found in the regions identified by Pritchard (J.P.), Stoneking (M.S.), and Sabeti (P.S.) [18], [54], [55]. (PDF) [file pgen.1003959.s015.pdf]

Natural Selection					POP 2							
					CHB				YRI			
POP 1	Source Author	Relevant POP	Genes	Gene Info	# Loci	POP 1 Variants	POP 2 Variants	P-value	# Loci	POP 1 Variants	POP 2 Variants	P-value
CEU	M.S.	EUR	LCT	Lactase	2258	8164	17181	7.255E-07	4133	10618	34844	1.314E-26
	J.P.	EUR	LCT		75	158	1088	1	185	602	1129	1
	P.S.	EUR	LCT		697	1413	7863	1.505E-03	1207	3064	11430	4.447E-22
	J.P.	AFR	ABCC11	Morpho. Traits	277	3478	150	1.096E-17	303	432	1420	6.173E-10
	J.P.	ASN	EDAR		360	1524	1184	2.710E-02	574	1226	4264	1.828E-19
	P.S.	ASN	EDAR		134	4462	1216	2.432E-26	168	140	766	1.388E-14
	M.S.	EUR	SLC24A5		987	1826	6736	7.382E-18	1604	2670	10047	3.686E-24
	J.P.	ASN	SLC24A2		1042	5522	3838	1	1647	2777	10374	1.753E-24
	J.P.	EUR	DUOX2	Immun. Respns.	44	115	75	1	130	86	1075	2.319E-26
	J.P.	EUR	ALMS1	Insulin Reg	780	8624	688	5.052E-08	1116	7977	2043	2.001E-24
	P.S.	EUR	ALMS1		544	5734	568	5.346E-04	803	1502	5947	5.060E-23
	J.P.	ASN	ADH1B	Metab. Reg.	41	223	190	1	77	167	455	6.642E-05
	J.P.	EUR	CPSF3L	Misc. / Unk.	46	135	241	8.589E-01	92	145	688	1.274E-16
	J.P.	EUR	FAIM		64	1250	462	9.653E-05	127	249	934	2.902E-24
	M.S.	ASN	LIMCH1		1584	7267	3567	3.297E-06	1083	2067	6123	1.059E-20
	J.P.	EUR	PCGF1		7	8	55	2.905E-06	14	8	55	3.756E-04
	J.P.	AFR	RNF135		83	183	75	7.000E-05	157	284	4311	2.142E-26
	J.P.	EUR / ASN	SLC30A9		323	2483	525	4.786E-11	541	1464	3847	1.991E-20
	M.S.	ASN			735	9261	1204	1.547E-10	1083	4509	6435	5.799E-02
	P.S.	AFR			112	2191	253	4.786E-11	131	883	458	9.199E-09
	J.P.	EUR	TTC31		29	63	25	1	51	28	303	2.072E-18
YRI	M.S.	EUR	LCT	Lactase	4179	26603	14402	7.397E-12				
	J.P.	EUR	LCT		215	1123	1293	1				
	P.S.	EUR	LCT		1247	7633	6310	1				
	J.P.	AFR	ABCC11	Morpho. Traits	420	8166	306	3.249E-18				
	J.P.	ASN	EDAR		601	3828	1016	6.360E-18				
	P.S.	ASN	EDAR		174	2916	124	7.385E-23				
	M.S.	EUR	SLC24A5		1483	8190	4216	2.789E-11				
	J.P.	ASN	SLC24A2		1729	18627	4085	4.140E-23				
	J.P.	EUR	DUOX2	Immun. Respns.	125	1226	52	3.869E-26				
	J.P.	EUR	ALMS1	Insulin Reg.	1239	28301	1565	1.252E-25				
	P.S.	EUR	ALMS1		922	19783	1311	1.248E-25				
	J.P.	ASN	ADH1B	Metab. Reg.	62	213	22	1.469E-14				
	J.P.	EUR	CPSF3L	Misc. / Unk.	77	622	223	7.590E-08				
	J.P.	EUR	FAIM		130	1993	396	1.784E-24				
	M.S.	ASN	LIMCH1		1095	11207	1086	4.804E-25				
	J.P.	EUR	PCGF1		15	55	55	1				
	J.P.	AFR	RNF135		146	4112	172	3.003E-25				
	J.P.	EUR / ASN	SLC30A9		568	6538	1023	2.897E-24				
	M.S.	ASN			1301	27895	7627	1.388E-25				
	P.S.	AFR			194	6665	1947	8.165E-23				
	J.P.	EUR	TTC31		55	483	25	1.853E-17				
