# Supplementary material for: Low Frequency Variants, Collapsed Based on Biological Knowledge, Uncover Complexity of Population Stratification in 1000 Genomes Project Data
Source: PLoS Genet. 2013 Dec 26;9(12):e1003959. doi: 10.1371/journal.pgen.1003959 (PMC3873241; doi:10.1371/journal.pgen.1003959)
Supplement: Table S3 — Specific genes of interest with known allele frequency differences between ancestral populations. (PDF) [file pgen.1003959.s016.pdf]

	POP 2	CHB				YRI			
POP 1		N. Loci	POP 1 Variants	POP 2 Variants	P-value	N. Loci	POP 1 Variants	POP 2 Variants	P-value
CEU	LCT	140	114	1030	4.239E-10	213	143	1799	1.026E-22
	PAH	246	1520	168	2.008E-07	492	1670	2737	1
	CTCF	225	836	207	8.828E-05	323	801	2412	4.129E-04
	CFTR	487	3340	428	4.536E-11	731	1737	4355	1.659E-16
YRI	LCT	214	1424	788	5.875E-01				
	PAH	530	4764	2892	2.099E-05				
	CTCF	311	3700	429	2.081E-21				
	CFTR	792	7396	1544	4.995E-23				
