# Supplementary material for: Low Frequency Variants, Collapsed Based on Biological Knowledge, Uncover Complexity of Population Stratification in 1000 Genomes Project Data
Source: PLoS Genet. 2013 Dec 26;9(12):e1003959. doi: 10.1371/journal.pgen.1003959 (PMC3873241; doi:10.1371/journal.pgen.1003959)
Supplement: Table S4 — Example of BioBin parameter options, rare-case-control (RCC) and overall-major-allele (OMA). Table S4 contains iterations of major/minor allele selection and variant binning using new parameters rare-case-control (RCC) and overall-major-allele (OMA). Using both options is necessary to make the results independent of control group selection. (PDF) [file pgen.1003959.s017.pdf]

Example	POP	Minor Allele	Major Allele	Group A	RCC ON	OMA ON	Minor Allele	Group A MAF	Low frequency (0.05)	Bin Count (GroupA)	Bin Count (GroupB)
A	CEU<br>YRI	T: 60<br>A: 10	A: 140<br>T: 190	CEU	NO	NO	T	0.3	NO		
B	CEU<br>YRI	T: 60<br>A: 10	A: 140<br>T: 190	YRI	NO	NO	A	0.05	YES	10	140
C	CEU<br>YRI	T: 60<br>A: 10	A: 140<br>T: 190	CEU	YES	NO	T	0.3	YES	60	190
D	CEU<br>YRI	T: 60<br>A: 10	A: 140<br>T: 190	YRI	YES	NO	A	0.05	YES	10	140
E	CEU<br>YRI	T: 60<br>A: 10	A: 140<br>T: 190	CEU	NO	YES	A	0.7	NO		
F	CEU<br>YRI	T: 60<br>A: 10	A: 140<br>T: 190	YRI	NO	YES	A	0.05	YES	10	140
G	CEU<br>YRI	T: 60<br>A: 10	A: 140<br>T: 190	CEU	YES	YES	A	0.7	YES	140	10
H	CEU<br>YRI	T: 60<br>A: 10	A: 140<br>T: 190	YRI	YES	YES	A	0.05	YES	10	140
