# Supplementary material for: Low Frequency Variants, Collapsed Based on Biological Knowledge, Uncover Complexity of Population Stratification in 1000 Genomes Project Data
Source: PLoS Genet. 2013 Dec 26;9(12):e1003959. doi: 10.1371/journal.pgen.1003959 (PMC3873241; doi:10.1371/journal.pgen.1003959)
Supplement: Table S5 — Excerpt of custom region file containing regions with signatures of natural selection. (PDF) [file pgen.1003959.s018.pdf]

Chrom	ID	Start(bp)	Stop(bp)
chr1	CMS_EUR:reg1	1490074	1509034
chr1	CMS_ASN:reg2	16414784	16417992
chr1	CMS_AFR:reg3	26917014	26936774
chr1	CMS_EUR:reg4	30707291	30724056
