# Supplementary material for: Low Frequency Variants, Collapsed Based on Biological Knowledge, Uncover Complexity of Population Stratification in 1000 Genomes Project Data
Source: PLoS Genet. 2013 Dec 26;9(12):e1003959. doi: 10.1371/journal.pgen.1003959 (PMC3873241; doi:10.1371/journal.pgen.1003959)
Supplement: Table S6 — Low frequency simulation using SimRare to assess Type I error and power. (PDF) [file pgen.1003959.s019.pdf]

Sample Size	Type I Error Rate	Power
2000	0.0538	0.9910
1000	0.0495	0.9340
500	0.0550	0.7575
250	0.0545	0.5030
