# Supplementary material for: Low Frequency Variants, Collapsed Based on Biological Knowledge, Uncover Complexity of Population Stratification in 1000 Genomes Project Data
Source: PLoS Genet. 2013 Dec 26;9(12):e1003959. doi: 10.1371/journal.pgen.1003959 (PMC3873241; doi:10.1371/journal.pgen.1003959)
Supplement: Text S1 — Differential bias in 1000 Genomes Project data. (DOCX) [file pgen.1003959.s020.docx]

# Supplemental Text 1. Differential bias in 1000 Genomes Project data

In a recent publication by the 1000 Genomes Project, the authors declared sequence errors to be relevant to the technology used but not to have any correlation with population identity. In order to determine if there was differential bias across populations or continental groups based on sequence technology, we performed principal component analyses in each continental group and reviewed the global variation differences in the context of sequence technology (similar approach to recent 1000 Genomes Consortium paper). We also reviewed the sequence technologies used for each population in the Phase I release (see Table S1)[11]. Before removing the 75 cryptically related individuals, only the TSI population was sequenced on a single technology. However, after dropping cryptically related individuals, CHB, CHS, and JPT were also sequenced exclusively with Illumina technology.

In Figure S1, we plotted the first two principal components calculated from each of the four continental groups shown in Table S1. We colored the scatter plots using population identity and then sequence technology. From Figure S1, it is clear that within continental groups, the largest source of variation is sequence technology. In all four groups, the first principal component perfectly separates based on technology. However, the variation does not also coincide with population identity and there is overlap between populations since few populations were sequenced on a single technology. This reduces the likelihood that sequence technology causes differential bias in the resulting trends of our analyses.

In Figure S2, we conducted a sample analysis binning rare variants by Entrez gene boundaries using a binning MAF threshold of < 5%. In order to account for technology covariates and the potential for complete separation by the covariates, we used Firth logistic regression [56] as the statistical test of choice. We see in Figure S2A that the overall stratification structure is very similar to that seen elsewhere in the paper, so we expect this to be analogous to all of the analyses seen in the paper.

The first method of correcting for technology bias involves the use of principal components, which is a standard method of correcting for stratification [57,58]. However, in a naïve implementation of principal component analysis, the population stratification dominates the technology effect, so we use a novel approach where we calculate the principal components two ways and then use a method of projection to eliminate the stratification caused by technology. Using this method, we can see in Figure S2B that the sequencing technology does not contribute to the observed stratification ion any meaningful way.

In Figure S2C, we use a more traditional method of correcting for technology effects by using the sequencing technology itself as covariates in the logistic regression. In this analysis, the technology was encoded using up to three binary dummy variables. We can see again that while the proportion of significant bins is slightly reduced, the effect is marginal. From this sample analysis, we can conclude that while the sequencing technology can cause a noticeable effect, this difference is orthogonal to the population stratification shown throughout the paper.

56. Heinze, G and Schemper, M (2002) A solution to the problem of separation in logistic regression. Statist Med 21: 2409-2419. doi: 10.1002/sim.1047.

57. Price AL, Patterson NJ, Plenge RM, Weinblatt ME, Shadick NA, Reich D (2006) Principal components analysis corrects for stratification in genome-wide association studies. Nat Gen 38(8): 904-909. doi:10.1038/ng1847.

58. Patterson N, Price AL, Reich D (2006) Population structure and eigenanalysis. PLoS Genet 2(12): e190. doi:10.1371/journal.pgen.0020190.
