# Supplementary material for: Low Frequency Variants, Collapsed Based on Biological Knowledge, Uncover Complexity of Population Stratification in 1000 Genomes Project Data
Source: PLoS Genet. 2013 Dec 26;9(12):e1003959. doi: 10.1371/journal.pgen.1003959 (PMC3873241; doi:10.1371/journal.pgen.1003959)
Supplement: Text S2 — Investigation of allele sharing in 1080 individuals. (DOCX) [file pgen.1003959.s021.docx]

# Supplemental Text 2. Investigation of allele sharing in 1080 individuals

## IBD estimation in common variants

In most genomic studies, subject relatedness is calculated using common variants (GWAS). Therefore, in the first allele sharing analysis, we estimated IBD using only common variants (MAF > 10%) in all 1080 individuals available in the Phase I release. In Figure S3, the y-axis and x-axis correspond to the proportion of markers identical by descent between a pair of individuals sharing one allele versus none. One can identify small clusters of individuals in the top left quadrant that correspond to more allele sharing than one would expect from unrelated individuals. For example, the green dots in the left plot of Figure S3A represent a subset of approximately 10 related individuals in the LWK population. Each dot represents the IBD estimate between two LWK individuals. The pairs that share one allele at almost 100% of possible loci (Z1 ~ 1) and share at least one allele at all loci (Z0 ~ 0) represent a parent child relationship. Siblings cluster near the center of the plot, and pairwise IBD estimates for completely unrelated individuals cluster in the lower right quadrant. In Figure S3, there are several “within population” plots that show increased allele sharing within population groups (A,C,E,G). However, there does not appear to be any increased sharing between population groups (B,D,F,H). For example, even though there are individuals in the LWK population that appear to be related (see Figure S3A), none of the three African descent populations appear to have closely related individuals across populations (i.e. LWK-YRI are not related, Figure S3B).

There were 16 LWK individuals with a proportion of IBD greater than 0.5 and evidence of first and second-degree relationships. Two of the four top related IBD pairwise comparisons in LWK have been calculated in other studies as parent-child relationships[25]. Most of the apparent relationships in the IBD plots above have been identified previously and are available on the 1000 Genomes Project website [http://www.1000genomes.org/phase1-analysis-results-directory, *cryptic relation analysis*]. Note: this was our preliminary IBD analysis; the IBD analysis used to eliminate cryptically related individuals is described in main text.

## IBS calculation in low frequency and common variants

A second method was used to evaluate allele sharing, this method has been described by Abecasis et al [12,13]. Utilizing plink-seq, identity-by-state (IBS) values were calculated for low frequency variants (MAF < 3%) and common variants (MAF > 25%) within and between continental groups. Again, there was increased sharing among ASW, CHB, CHS, CLM, GBR, JPT, LWK, and MXL populations. It is very likely there are at least extended relatives in these populations.

Figure S4 shows the mean IBS calculations (averaged across 22 autosomal chromosomes) in low frequency variants for all pairwise individuals within a continental ancestry group. The left plot (Figure S4A) corresponds to the IBS calculations for all 1080 individuals; the right plot (Figure S4B) shows the IBS calculations after removing cryptically related individuals. The X-axis corresponds to the index number comparison; each x index value represents one pairwise comparison.  The comparisons are grouped and colored by type (i.e. CHS-CHS and CHS-JPT).  The Y-axis corresponds to the mean IBS calculation across all 22 autosomal chromosomes.  In Figure S4, low IBS means correspond to very little allele sharing for variants with MAF < 0.03.  Higher IBS means correspond to more allele sharing (and perhaps relatedness) among individuals in that pair. For example, there is increased sharing of alleles with < 3% MAF among LWK pairs (teal peaks, Figure S4A).

Common variant IBS calculations alone overestimate IBD, but we wanted to repeat the analysis shown in Figure S4 for common variants. We repeated the IBS calculations using only variants with a continental group minor allele frequency of 25% or higher. In Figure S5, the left plot shows the IBS calculations in all 1080 individuals; the right plot shows the IBS calculations after removing 75 cryptically related individuals. We see the same peaks of increased sharing in LWK, ASW, GBR, CHS, and MXL and the removal of those cryptically related individuals reduces the amount of sharing in those populations.
