# Supplementary material for: Low Frequency Variants, Collapsed Based on Biological Knowledge, Uncover Complexity of Population Stratification in 1000 Genomes Project Data
Source: PLoS Genet. 2013 Dec 26;9(12):e1003959. doi: 10.1371/journal.pgen.1003959 (PMC3873241; doi:10.1371/journal.pgen.1003959)
Supplement: Text S3 — Additional results from regions of natural selection analysis. (DOCX) [file pgen.1003959.s022.docx]

# Supplemental Text 3. Additional results from regions of natural selection analysis

Regions of natural selection have been identified using various methods often in a population specific manner. Therefore, we expect large differences in low frequency variant burden between populations that do not share similar selective patterns. Using the 1005 individuals, we investigated specific genes provided by Barreiro et al. that have been found to show the strongest signatures of positive selection. These genes contain at least one nonsynonymous or 5’ UTR mutation with an FST value greater than 0.65[19].

Using CEU/CHB/YRI populations as representative populations from the European, Asian, and African ancestral groups, the regions of natural selection from three published sources associated with the gene list provided by Barreiro are shown in Table S2 [18,54,55]. This table includes the number of loci in the bin, total binned variants from both populations, and the bin p-value. The source author corresponds to the paper for that particular region. The “relevant population” describes the population where the signature of selection was found.

Next, we were interested in taking a closer look at particular genes known to have allele frequency differences between populations: Lactase, Phenylalanine Hydroxylase, CTCF, CFTR. Table S3 shows the BioBin p-value results for three representative populations (YRI, CEU, CHB) in each gene bin.  For example, the Lactase gene had a significant low frequency variant burden difference between both CEU/YRI and CEU/CHB but not YRI/CHB. On the other hand, CFTR was significantly different in all three comparisons.
