# Supplementary material for: Low Frequency Variants, Collapsed Based on Biological Knowledge, Uncover Complexity of Population Stratification in 1000 Genomes Project Data
Source: PLoS Genet. 2013 Dec 26;9(12):e1003959. doi: 10.1371/journal.pgen.1003959 (PMC3873241; doi:10.1371/journal.pgen.1003959)
Supplement: Text S4 — Additional binning algorithm details. (DOCX) [file pgen.1003959.s023.docx]

# Supplemental Text 4. Additional binning algorithm details

The analysis approach used with BioBin proceeds in two steps: first, BioBin generates bins based on user-defined parameters and information from LOKI; second, the user applies an appropriate statistical association test. To bin, the user can choose options in the configuration file to select certain database sources, adjust feature types (such as gene, pathway, regulatory regions, etc), and/or configure the minor allele frequency (MAF) binning threshold. The MAF binning threshold determines the allele frequency limit under which variants are binned.

Throughout this analysis, we refer to the term locus as a strict chromosome coordinate or position. The term variant corresponds to the alleles at that locus. In the 1000 Genomes Project Phase I population comparison, there is no case or control group, instead we will use the terms “Group A” and “Group B” which are analogous to cases and controls in a typical association analysis. Two improvements were added to BioBin methodology to better account for population comparison data. The first option addresses how BioBin handles the minor allele frequency-binning threshold in two groups. In a typical case-control analysis, the user likely wants to bin variants at each locus that fall under the minor allele frequency-binning threshold in controls (i.e. only bin if low frequency in controls). However, in a population comparison, if the variant is low frequency in EITHER group, the LOCUS should collapse into a bin. In Table S4, this option is labeled Rare-Case-Control or “RCC”. This does not change which allele is considered the minor allele, the minor allele is annotated using Group A allele frequencies, but it does include specific sites (or loci), which have a low frequency variant in Group B (but not in Group A). In Table S4, each line corresponds to the way a variant would be handled under various permutations of these options for 100 CEU individuals (MAF=0.3) and 100 YRI individuals (MAF=0.05). Examples A-D show the function of the rare-case-control option. In example A, the locus is not rare enough in Group A to be binned. Under the same parameter conditions, if other population is used as Group A, the locus is binned because it meets the minor allele frequency threshold. It is obviously not beneficial for results to change based on Group A in pairwise population comparisons. Examples C-D show how the locus is binned regardless Group A with the “RCC” option is used. The minor allele frequency within each group doesn’t change and therefore the allele chosen as the minor allele is still dependent on Group A, but a locus can be binned if it is “rare” in either group. We found that when RCC is OFF at low binning thresholds, the number of loci in a bin is highly correlated with significance. Using the RCC option eliminates the bin size correlation with significance.

The second parameter option concerns choosing the minor allele. Typically, the minor allele at a given locus is determined from the second most frequent allele in the control group. However, in the 1000 Genomes population comparison, we found that switching the group status changed the results by 1-3% because the specified minor allele at a locus could change based on which population was designated as the controls. The option to address this problem is labeled “overall-major-allele”, denoted as “OMA” in the Table S4. This option was added to allow BioBin to look at Group A and Group B (i.e. cases and controls) before determining which alleles are the major and minor alleles. When the overall-major-allele option is turned on, the major allele is designated by the overall highest frequency allele (considering Group A and Group B). Examples E-H in Table S4 show the benefits of using this additional option. Specifically, comparing the low frequency variant count between Group A and Group B, examples C-D show more similar counts than A-B, but G-H have the exact variant counts which means that the choice of Group A is unimportant. Using rare-case-control and overall-major-allele options requires BioBin to review allele counts collectively between the two groups to choose the minor allele. The individual group minor allele frequency does not change, but which allele is considered the major (and thus minor) allele can change. The overall minor allele is not necessarily the Group A minor allele. As shown in examples G-H, this is the ideal condition for a population comparison where results should be independent of which group is chosen as Group A (or the control).

Again, for this 1000 Genomes Project population comparison, the case/control status does not have a true meaning. Without RCC = ON and Overall = ON, the results can change based on which group is designated as the Group A or the control group. In other situations, such as a case-control study, these options should be carefully chosen based on study size, MAF binning threshold, and study design.

## Example custom region file

Custom feature files can be used in place of or in addition to LOKI knowledge. An excerpt of the custom region file used for the natural selection feature is shown in Table S5. The first column corresponds to the chromosome and the second column is the region ID. The regions of natural selection were named according to related genes, pertinent population where selection signatures were found, and the last author of the publication that identified this region. The third and fourth columns correspond to the start and stop positions of the custom region.

## Inter-region bins

After feature selection using BioBin and/or external custom files, inter-region bins can be created. Inter-region bins catch variants that do not fit into the user-defined feature types. For example, if one were testing low frequency burden differences between Group A and Group B across genes, all variants in genes would be collapsed into respective gene bins, and variants outside of gene boundaries would be binned corresponding to the intergenic regions. BioBin provides an option to generate inter-region bins of a user-specified size to catch inter-region variants. Figure 8 in the main text shows an example of low frequency binning strategies for 12 variants that have fallen below the MAF binning threshold. The strategy binning examples show how different knowledge applied to the same variants produces alternate bins.
