# Supplementary material for: Low Frequency Variants, Collapsed Based on Biological Knowledge, Uncover Complexity of Population Stratification in 1000 Genomes Project Data
Source: PLoS Genet. 2013 Dec 26;9(12):e1003959. doi: 10.1371/journal.pgen.1003959 (PMC3873241; doi:10.1371/journal.pgen.1003959)
Supplement: Text S5 — Data simulation strategy. (DOCX) [file pgen.1003959.s024.docx]

# Supplemental Text 5. Data simulation strategy

As BioBin is a novel approach, we utilized simulations to test BioBin. Genetic data was simulated using SimRare[59], a GUI interface simulation program built around a forward time simulator, simuPOP[60]. simuPOP can simulate introduction and evolution of rare variants and can allow complex fitness and selection modeling (http://simupop.sourceforge.net, https://code.google.com/p/simrare/). There are a few reasons SimRare is preferable to simuPOP: it is computationally efficient, time efficient, and reduces the number of linking scripts needed to create many replicates of simulated data.

We used an additive multilocus selection model with a selection distribution described by Kryukov[61]. The mutation rate was set at of 1.8 x 10^-8^ per nucleotide per generation. The population sizes were Ne = 8100, 8100, 7900, and 900000 with 5000 generations, 10 generations, and 370 generations respectively. A fixed 5kb region of genetic data was simulated on the same evolutionary parameters for 250 replicates. In this instance the term replicate is a realization of the forward-time simulation using the given input parameters. Of course, each replicate is not necessarily the same since evolution cannot be exactly repeated due to randomness. However, more replicates slightly increase the diversity of the final simulated data. Each simulated population randomly samples the replicates (hence the increased diversity) for a given population size and applies the indicated genetic model (i.e. null model or a model fitting particular effect sizes). In the simulation studies below, we sampled 4000 times (R) at several population sizes (N=2000, 1000, 500, 250) and applied the genetic models to test type I error and power. For each BioBin run, the entire simulated region was considered a single bin.

To generate a sample data set evaluating type I error, the following parameters were used: 1.0 odds ratio for protective and detrimental mutations and an additive mode of inheritance. We did not incorporate missingness or unphenotyped individuals. The case/control status was evenly and randomly assigned. We calculated the type I error rate as the number of bins with Wilcoxon p-value less than or equal to 0.05 divided by the total number of bins simulated (R). An error rate above 5% would indicate a higher false-positive test and an error rate lower than 5% would indicate a conservative test.

To generate a sample data set evaluating power, the following parameters were used: 0.9 odds ratio for protective mutations, 2.5 odds ratio for detrimental mutations, and an additive mode of inheritance. Again, we did not incorporate missingness or unphenotyped individuals. The power was calculated as the proportion of R bins, where R is number of simulated bins, with a p-value <= 0.05.

As shown in Table S6, the Wilcoxon 2-sample rank sum test was slightly anticonservative, but seems independent of sample size. The type I error rate remained very close to 0.05 at each population size. The power was greater than 90% at sample sizes greater than 1000. The power drops to 75% at a sample size of 500 and 50% at a sample size of 250. Although the power drops quickly with sample size, this is still not much of a concern in this particular analysis. An odds ratio of 2.5 is quite conservative for low frequency variants. The signal in the population comparison data will be much larger due to the great diversity of allele frequencies and population stratification in the low frequency variants.

59. Li B, Wang G, Leal SM (n.d.) SimRare: a program to generate and analyze sequence-based data for association studies of quantitative and qualitative traits. Bioinformatics. Available: http://bioinformatics.oxfordjournals.org/content/early/2012/08/22/bioinformatics.bts499. Accessed 26 January 2013.

60. Peng B, Amos CI, Kimmel M (2007) Forward-time simulations of human populations with complex diseases. PLoS Genet 3: e47. doi:10.1371/journal.pgen.0030047.

61. Kryukov GV, Shpunt A, Stamatoyannopoulos JA, Sunyaev SR (2009) Power of deep, all-exon resequencing for discovery of human trait genes. Proc Natl Acad Sci U S A 106: 3871–3876. doi:0812824106 [pii];10.1073/pnas.0812824106 [doi].
